# Apomorphine is a novel necroptosis inhibitor targeting mixed lineage kinase domain-like protein oligomerization

**DOI:** 10.1038/s41420-025-02763-8

**Published:** 2025-10-13

**Authors:** Myeonggil Han, Dong-Hyun Seo, Man Sup Kwak, In Ho Park, Woo Joong Rhee, Hee Sue Kim, Eunkyeong Jeon, Je-Jung Lee, Cheol Ho Park, Nam Doo Kim, Taebo Sim, You-Sun Kim, Kyoung-Seok Ryu, Jeon-Soo Shin

**Affiliations:** 1 Department of Microbiology, Yonsei University College of Medicine, Seoul, South Korea; 2 Brain Korea 21 FOUR Project for Medical Science, Yonsei University College of Medicine, Seoul, South Korea; 3 Ochang Center, Korea Basic Science Institute, Cheongju-Si, South Korea; 4 KBSI School of Bioscience, University of Science and Technology, Daejeon, South Korea; 5 Institute for Immunology and Immunological Diseases, Yonsei University College of Medicine, Seoul, South Korea; 6 Department of Biomedical Sciences, Yonsei University College of Medicine, Seoul, South Korea; 7 Voronoibio Inc., Yeonsu-gu, South Korea; 8 Department of Biochemistry, Ajou University School of Medicine, Yeongtong-gu, Republic of Korea; 9 Department of Biomedical Sciences, Graduate School of Ajou University, Yeongtong-gu, Republic of Korea; 10 Center for Nanomedicine, Institute for Basic Science (IBS), Yonsei University, Seoul, South Korea

**Keywords:** Necroptosis, Drug screening, Inflammation

## Abstract

Necroptosis, a form of programmed cell death, has emerged as a promising therapeutic target. Although several RIPK1 inhibitors have demonstrated favorable safety profiles in clinical trials, clinical translation of necroptosis-targeted therapies remains limited by modest efficacy, limited specificity, and species-specific activity of compounds such as necrosulfonamide (NSA). To resolve these challenges, this study identified a potential necroptosis inhibitor from a clinical drug library. Apomorphine (APO), a non-addictive morphine derivative used to treat Parkinson’s disease, was found to inhibit necroptosis by sterically blocking key residues involved in mixed lineage kinase domain-like protein (MLKL) activation and oligomerization, as confirmed by nuclear magnetic resonance analysis. APO is redox sensitive and prone to auto-oxidation. The oxidized form of APO (Ox-APO) showed stronger binding to MLKL than the reduced form of APO (Re-APO), as demonstrated by surface plasmon resonance analysis. Ox-APO significantly ameliorated tissue damage in two murine necroptosis models: dextran sulfate sodium (DSS)-induced colitis and acetaminophen (APAP)-induced liver injury. Collectively, these data highlight the therapeutic potential of APO as a necroptosis-specific inhibitor in necroptosis-related diseases in both humans and mice.

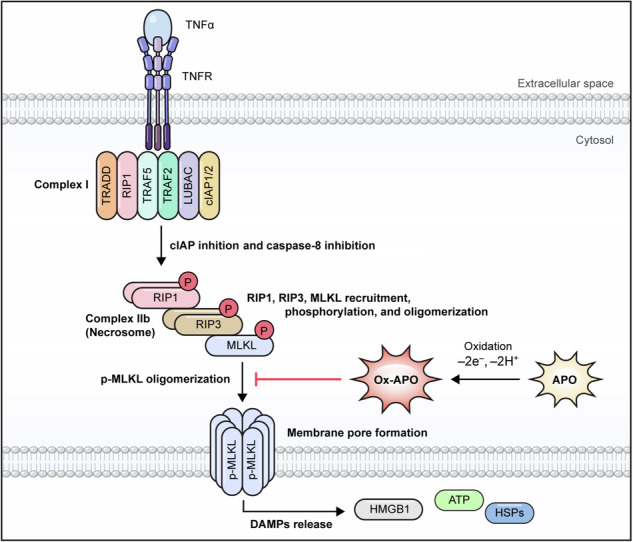

## Introduction

Necroptosis is characterized by cell swelling, plasma membrane rupture, and secretion of danger-associated molecular pattern molecules (DAMPs) [1–3]. Necroptosis is dependent on the activation of receptor-interacting protein kinase (RIP)1 and/or RIP3, resulting in the formation of a necrosome complex that initiates a cell death signaling cascade. Once formed, the necrosome triggers a downstream signaling cascade that activates mixed lineage kinase domain-like protein (MLKL) [4], the final and critical effector molecule of necroptosis. When MLKL molecules are phosphorylated, their oligomeric complexes formed in the cytoplasm are translocated to the plasma membrane to form necroptotic pores that disrupt membrane integrity [5]. In RIP1-independent necroptosis, Toll/interleukin-1 receptor (TIR) domain-containing adaptor-inducing IFN-β (TRIF) and Z-DNA binding protein 1 (ZBP1) bind to RIP3 and mediate necroptosis [6].

Clearance of necrotic cells is a complex process involving the recruitment of phagocytic cells to engulf and remove dying cells [7]. Secreted molecules, including DAMPs, induce an inflammatory response [8]. High mobility group box 1 (HMGB1), a representative DAMP molecule, is known to cause various inflammation-related diseases when excessively secreted during acute or chronic inflammatory responses [9–11]. The necroptosis pathway has been implicated in multiple disorders, such as systemic inflammation [12], viral infection [13], liver injury [14], myocardial infarction [15], atherosclerosis [16], retinal degeneration [17], cancer [18], and inflammatory bowel disease (IBD) [19]. IBD is a highly prevalent inflammatory condition affecting the gastrointestinal tract [20], whereas liver injury involves various forms of hepatocyte death [21]. Recently, a strong correlation between necroptosis and both IBD and liver injury has been demonstrated by knock-out models of necroptosis mediators by targeting necroptosis-related proteins such as RIP1, RIP3, MLKL, and DAMPs [22–24], along with histological assessments [25, 26] and measurements of enzyme and cytokine secretion [27]. Several small inhibitor molecules have been identified that target different molecules in the necroptotic pathway, including RIP1, RIP3, and MLKL [28]. For example, necrostatin-1 (NEC-1) is a specific inhibitor of RIP1, which initiates necroptosis [1]. GSK'872 and HS-1371 are selective inhibitors of RIP3 kinase activity, preventing MLKL phosphorylation and necroptosis pore formation [29, 30]. Necrosulfonamide (NSA) targets the ATP-binding pocket of human MLKL and prevents its oligomerization and translocation to the plasma membrane [4]. However, NSA exhibits human-specific activity and is ineffective against mouse MLKL [31], limiting its preclinical utility. Although several RIPK1 inhibitors under development have demonstrated favorable safety profiles in clinical trials [32, 33], their clinical efficacy has been mixed. For instance, GSK2982772, the RIPK1 inhibitor tested in humans, was well tolerated but failed to show significant therapeutic benefit in a phase II trial for ulcerative colitis [34]. Similarly, the RIPK1 inhibitor SAR443060 entered phase I/II trials for neurodegenerative diseases but was eventually discontinued due to long-term preclinical toxicology concerns [35]. These cases highlight the ongoing challenges in developing necroptosis inhibitors with robust efficacy and cross-species applicability, underscoring the need for safer and more broadly effective therapeutic strategies.

The object of this study was to identify a necroptosis inhibitor targeting the final effector molecule MLKL. We employed drug repurposing strategies and screened a comprehensive clinical chemical library consisting of 2150 compounds, including those in phase 1–3 clinical trials and FDA-approved drugs. We identified a novel necroptosis inhibitor, apomorphine (APO), which targets MLKL. APO is a derivative of morphine that does not bind to the opioid receptors responsible for drug addiction and has been used for the “off” effect in advanced Parkinson’s disease [36]. Notably, APO is redox sensitive and spontaneously oxidized to apomorphine (Ox-APO). Ox-APO efficiently inhibits cell necroptosis and effectively alleviates mouse IBD and liver injury symptoms of dextran sulfate sodium (DSS)-induced colitis and APAP-induced liver injury as a necroptosis model. Surface plasmon resonance (SPR) and nuclear magnetic resonance (NMR) studies of Ox-APO showed stronger binding to MLKL than APO and inhibited necroptosis pores. In conclusion, we found that APO, an FDA drug for Parkinson’s disease, is a newly discovered necroptosis inhibitor targeting MLKL oligomerization, and Ox-APO showed better efficacy than the reduced form of APO (Re-APO), making it a promising candidate for the treatment of necroptosis-related diseases in the future.

## Results

### Screening of inhibitor candidates against the necroptosis signaling pathway

**Fig. 1:**
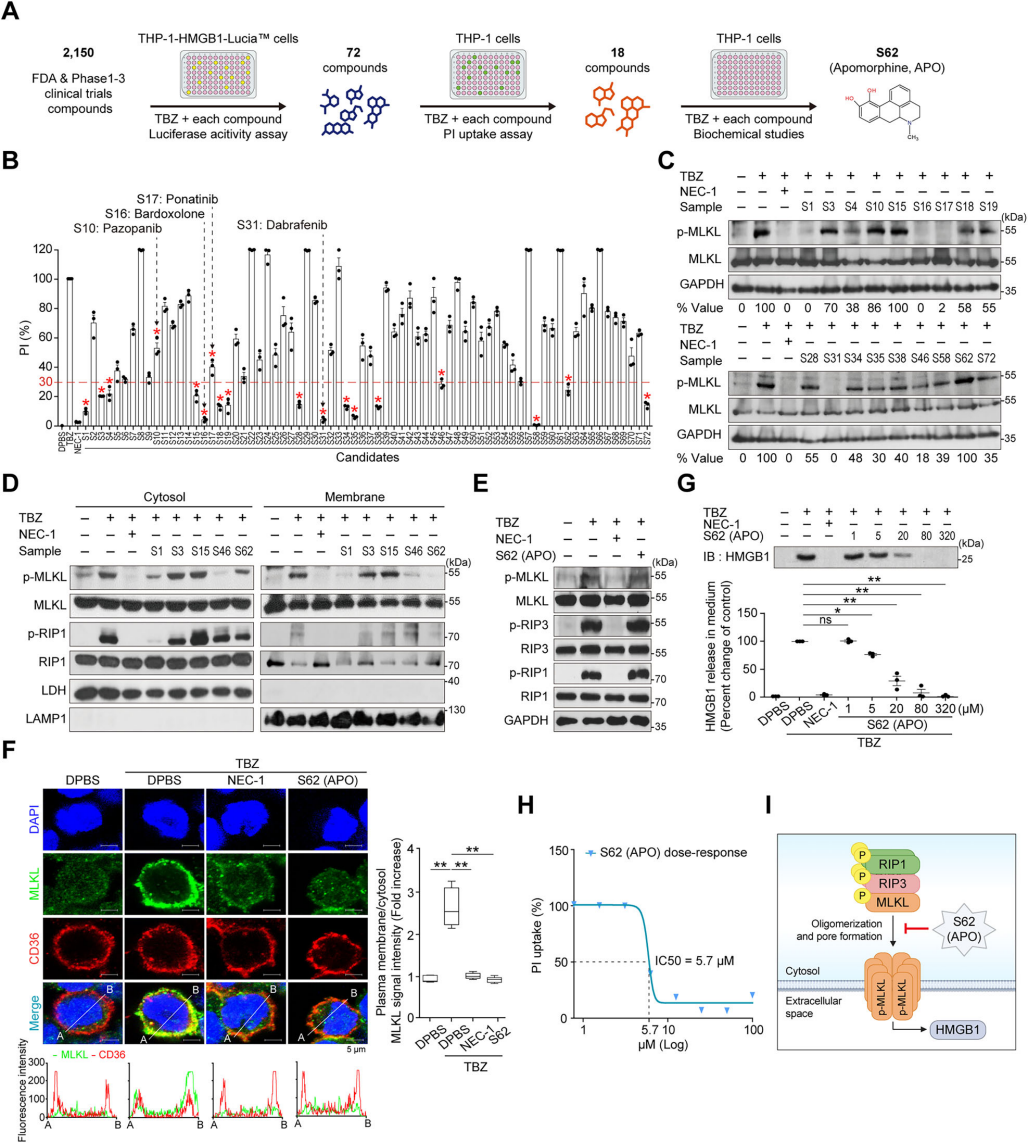
Screening and selection of necroptosis signaling inhibitor molecules. **A**, **B** Workflow of necroptosis inhibitor screening. THP-1-HMGB1-Lucia^TM^ cells (1 × 10^5^ cells/well) were treated with a combination of TBZ (a necroptosis inducer) and each chemical compound for 8 h to harvest the supernatants. Seventy-two compounds that inhibited luciferase activity by <20% compared to TBZ-treated cells were selected from the pool of 2150 compounds and then tested for PI uptake after TBZ and each compound treatment to select 18 compounds. The luciferase assay was repeated at least twice for selection. Eighteen compounds (*) were those that inhibited PI uptake signal <30% of TBZ-treated cells (red dotted line), and known necroptosis inhibitors (S10, S16, S17, and S31). Results are the mean of three independent experiments. NEC-1 is used as a positive control inhibitor. **C**, **D** THP-1 cells were treated with TBZ and each selected candidate for 8 h. Whole cell lysates (WCLs) were prepared for Western blot analysis to observe phosphorylated (p-) MLKL. The p-MLKL/MLKL ratio was quantified relative to TBZ-treated cells. Data represent the mean of three independent experiments. The quantification graph from three replicates is shown in Fig. S1A (**C**). THP-1 cells were separated into cytosol and membrane fractions, and p-MLKL and p-RIP1 were determined using MLKL and RIP1, respectively. LDH and LAMP1 were used as cytosolic and membrane markers, respectively. A representative blot is provided, and the quantification graph from three independent experiments is shown in Fig. S1B (**D**). **E** THP-1 cells were treated with TBZ and 20 μM S62, which is APO, for 8 h. WCLs were prepared to detect p-MLKL, RIP3, and RIP1 for immunoblotting. A representative experiment of three replicates is shown. **F** THP-1 cells were treated with TBZ and APO for 8 h and stained with DAPI, MLKL, and the membrane marker CD36 for confocal microscopy. Co-localization of MLKL with CD36 was analyzed to assess its membrane translocation. In the intensity graph, green indicates MLKL and red indicates CD36. The signal intensity of MLKL fluorescence from the plasma membrane to the cytosol was measured using FluoView FV1000 software. Mean ± SEM (*n* > 100). **p < 0.001, one-way ANOVA. Data re*p*resent three independent experiments with mean ± SEM (*n* = 3). **G** Inhibition of TBZ-mediated HMGB1 release by APO. THP-1 cells were treated with TBZ and different concentrations of APO for 8 h. HMGB1 levels were determined in the cell culture supernatant. Results are the mean of three independent experiments. *p < 0.05, **p < 0.001, ns not significant, one-way ANOVA. **H** THP-1 cells were treated with APO, and PI uptake was measured to estimate the IC_50_. The percentage of PI uptake level was compared with that of TBZ-treated cells. Results are the mean of three independent experiments. **I** Hypothetical mechanism of APO.

To identify potential inhibitors of the necroptosis pathway targeting MLKL or the process of p-MLKL pore formation on the membrane, we screened a library of 2150 compounds, including FDA-approved drugs and those in phase 1–3 clinical trials, using THP-1-HMGB1-Lucia™ cells expressing the HMGB1-luciferase fusion protein. The cells were treated with a necroptosis-inducing mixture containing TNF-α, BV6, and Z-VAD-FMK (TBZ) in addition to the compound library (Fig. 1A). The supernatants were harvested to measure luciferase activities for necroptosis. The luciferase activities of cell supernatants treated with TBZ alone were considered as the maximum release (100%) of HMGB1-luciferase. NEC-1, a known RIP1 inhibitor used in both humans and mice, was used as a positive control [37]. An initial screening was performed to identify compounds that reduced luciferase activity to within 20% of the TBZ-treated supernatant, and 72 compounds (S1–S72) were selected (Table S1). Among these compounds were four known necroptosis inhibitors of pazopanib (S10) [38], bardoxolone (S16) [39], ponatinib (S17) [38], and dabrafenib (S31) [40], indicating the reliability of this screening assay. Next, we performed a propidium iodide (PI) uptake assay to exclude cytotoxic compounds using THP-1 cells. Then, we selected candidate molecules that were arbitrarily less than 30% of those treated with TBZ. Eighteen compounds, including four known ones, were selected (Fig. 1B). Next, the inhibition levels of p-MLKL were tested to select MLKL-targeting molecules (Figs. 1C and S1A). Compounds that reduced p-MLKL levels to less than 30% of TBZ-treated controls were considered as potential inhibitors of MLKL phosphorylation. Conversely, compounds that maintained p-MLKL levels above 70% were selected because they are presumed to prevent the formation of pores in the plasma membrane by interfering with oligomerization [41]. Eventually, novel S1, S3, S15, S46, and S62 molecules were selected, excluding known inhibitors, and their effects on MLKL and RIP1 phosphorylation in cytosol and membrane fractions were examined (Figs. 1D and S1B). S3 and S15, which had almost no effect on p-MLKL levels in both cytosolic and membrane fractions, as well as S46 (Proscillaridin), a known apoptosis inducer [42], were excluded from further analysis. S1 (JP-1302), which seemed to inhibit RIP1 phosphorylation directly or indirectly, was also excluded.

**Fig. 2:**
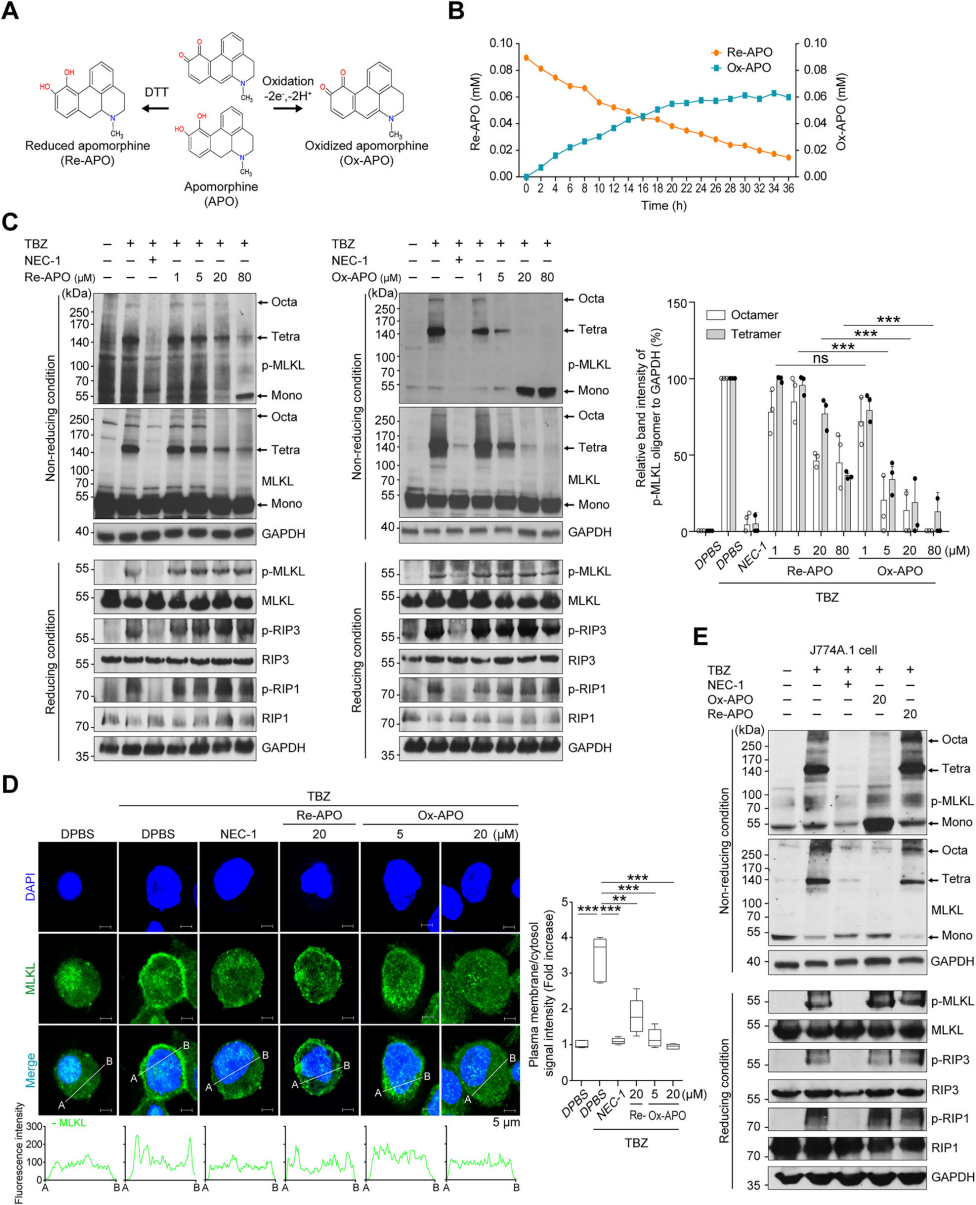
Effect of redox-sensitive APO on MLKL oligomerization. **A** Chemical structure of APO. APO undergoes auto-oxidation to form the oxidized form (Ox-APO), while the addition of DTT preserves the reduced form (Re-APO). The red highlights indicate the sites where APO loses hydrogen and electrons during the oxidation process. **B** Real-time 1D ^1^H NMR spectra showing the degree of oxidation of APO. APO was dissolved from its powdered form in DPBS and incubated at 25 °C for 36 h. Real-time 1D ^1^H NMR spectra were measured at a 2 h intervals after filtration through a 0.2 μm syringe filter to remove precipitates. The graph shows the formation of Ox-APO over time as Re-APO is oxidized in aqueous solution. **C** MLKL oligomerization is inhibited by APO. THP-1 cells were treated with TBZ and Re-APO or Ox-APO for 8 h. Western blot analysis of WCLs was performed under non-reducing and reducing conditions using the indicated antibodies. Percentage changes of p-MLKL oligomer band relative to GAPDH were shown. The *p* value for MLKL oligomer band intensity was calculated based on the average intensity of the tetramer and octamer bands. Octa octamer, Tetra tetramer, Mono monomer. Mean ± SEM (*n* = 3). **p < 0.001, ns not significant, one-way ANOVA. **D** THP-1 cells were treated with TBZ and Ox-APO or Re-APO for 8 h and then stained with DAPI and MLKL for confocal microscopy. In the intensity graph, green indicates MLKL. The signal intensity of MLKL fluorescence from the plasma membrane to the cytosol was measured using FluoView FV1000 software. Mean ± SEM (*n* > 100). **p < 0.01, ***p < 0.001, one-way ANOVA. **E** J774A.1 mouse monocytic cells were treated with TBZ and 20 μM Re-APO or Ox-APO for 8 h. Western blot analysis of WCLs was performed under non-reducing and reducing conditions using the indicated antibodies.

One remaining S62 (APO) significantly inhibited p-MLKL levels in the membrane fraction, although it showed little or moderate inhibition in the cytosolic fraction. We confirmed that APO showed little to no effect on p-MLKL, p-RIP3, and p-RIP1 in whole cell lysates (WCLs) (Fig. 1E). Confocal analysis revealed that APO treatment profoundly decreased the level of MLKL in the cell membrane, allowing it to be observed in the cytosol (Fig. 1F). The membrane marker CD36 was used for comparison. APO also dose-dependently decreased HMGB1 release in TBZ-treated THP-1 cells and also inhibited PI uptake after TBZ treatment (Fig. 1G, H), suggesting that APO effectively prevents necroptosis-induced membrane permeability. We hypothesized that APO is a candidate MLKL-dependent necroptosis inhibitor for pore formation independent of RIP1 and RIP3. APO is an oxidation-sensitive compound. APO was prepared in powdered form and was neither treated with a reducing agent nor incubated for oxidation during the initial screening. Next, we investigated the effect of the redox-sensitive APO on MLKL oligomerization (Fig. 2A).

### Effect of redox-sensitive APO on MLKL oligomerization

APO is prone to auto-oxidation in solution over time, especially in the presence of light and air, resulting in the formation of Ox-APO, which is characterized by a green color [43]. The addition of 1,4-dithiothreitol (DTT) can prevent the oxidation of APO, thereby maintaining its reduced form, Re-APO (Fig. 2A). When freshly dissolved APO was incubated at 25 °C for 36 h, real-time 1D ^1^H-NMR spectra showed a progressive increase in APO oxidation, reaching saturation at 24 h (Fig. 2B). This suggests that APO in solution may contain some Ox-APO. To demonstrate the effect of both Re-APO and Ox-APO on MLKL oligomerization, we prepared Re-APO and Ox-APO, which were treated with DTT and allowed to oxidize APO in an aqueous solution for 24 h, respectively. Western blotting showed that Ox-APO strongly inhibited the tetramerization and octamerization of p-MLKL in a concentration-dependent manner from the p-MLKL monomer under non-reducing conditions (Fig. 2C). In contrast, treatment with Re-APO was less effective than Ox-APO, and both Re-APO and Ox-APO had little effect on the phosphorylation levels of RIP1, RIP3, and MLKL. Confocal microscopy further demonstrated that Ox-APO was more effective than Re-APO in inhibiting the migration of p-MLKL to the cell membrane, and the punctate MLKL signals were observed in the cytosol at the same concentration (Fig. 2D). Next, we examined whether APO could prevent necroptosis by using J774A.1, a mouse monocytic cell line, for an in vivo mouse study. Western blotting revealed that both Ox-APO and Re-APO strongly prevented the formation of mouse MLKL (mMLKL) tetramers and octamers at 20 μM, similar to their effect on human THP-1 cells. Ox-APO showed a better effect (Fig. 2E). Similarly, the Re-APO and Ox-APO treatments had little effect on the phosphorylation of mRIP1, mRIP3, and mMLKL. All these results showed that APO primarily interferes with MLKL oligomerization.

To further validate that Ox-APO inhibits MLKL oligomerization-driven necroptosis, we used an MLKL-gyrase fusion construct that is induced by doxycycline (Dox) and undergoes oligomerization when treated with coumermycin (Fig. S2) [44]. In J774A.1 cells transfected with Wt-mMLKL (1–464)-gyrase, Ox-APO treatment markedly reduced PI-positive cells following Dox and coumermycin stimulation. These results support that Ox-APO suppresses necroptosis by binding directly to MLKL and preventing its oligomerization.

### SPR analysis of APO binding to MLKL

**Fig. 3:**
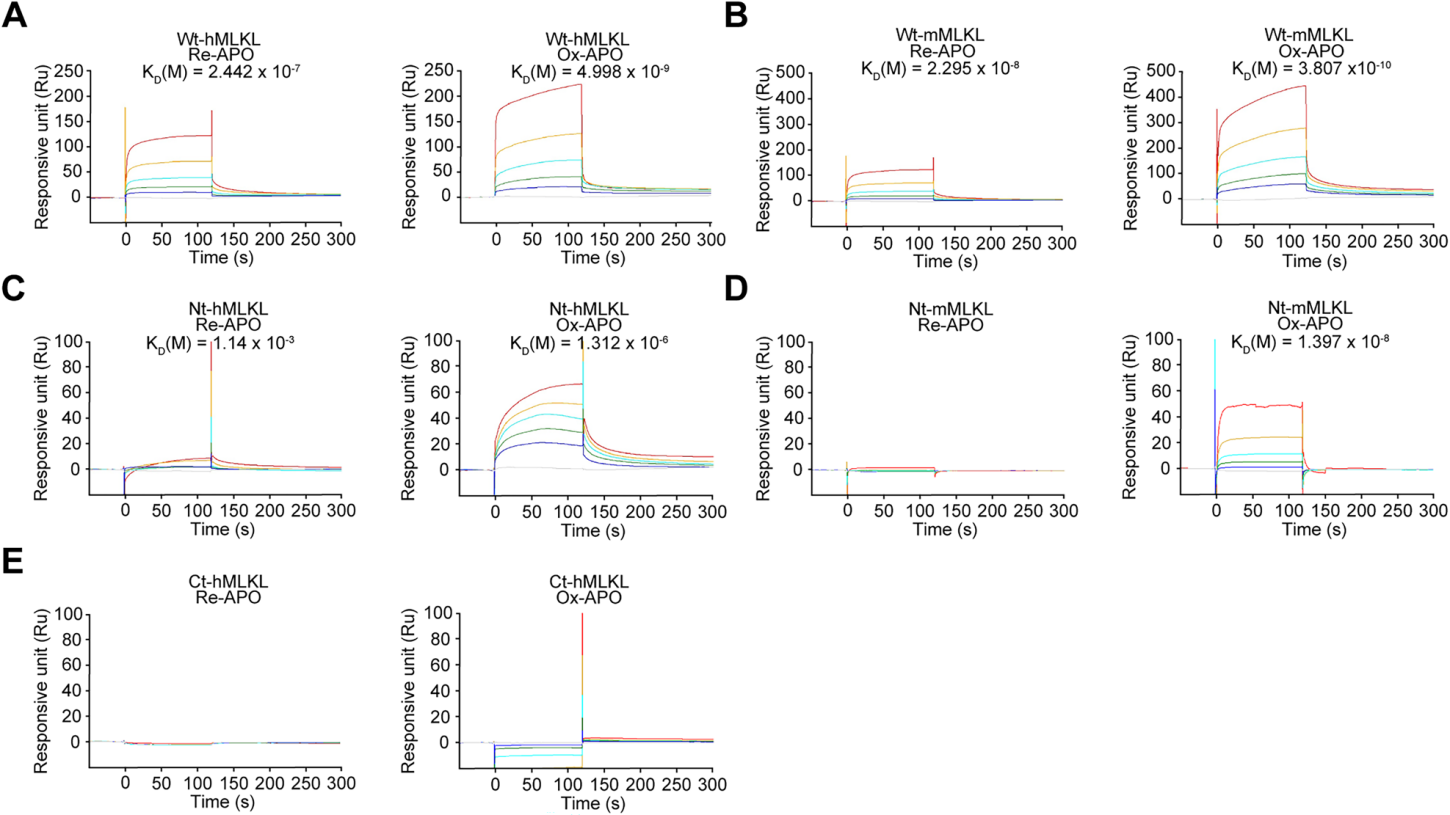
SPR analysis of APO to MLKL. **A**–**E** Human and mouse wild-type MLKL (Wt-hMLKL and -mMLKL), N-terminal (Nt) and C-terminal (Ct)-hMLKL, Nt-mMLKL proteins were immobilized on CM5 chips. Re-APO and Ox-APO analytes were added at concentrations of 0 μg/mL, 12.5 μg/mL, 25 μg/mL, 50 μg/mL, 100 μg/mL, and 200 μg/mL. *K*_D_ values were measured using BIAevaluation software.

The wild-type human MLKL protein (Wt-hMLKL) consists of two domains: an N-terminal domain (Nt-hMLKL, amino acids 2–154, MW: 23 kDa) involved in oligomerization and a C-terminal domain (Ct-hMLKL, amino acids 183–471, MW: 31 kDa) involved in phosphorylation [45]. To investigate the binding characteristics of Re-APO and Ox-APO with Nt-hMLKL, we performed SPR to evaluate the binding of Re-APO and Ox-APO to wild-type human and mouse MLKL (Wt-hMLKL, -mMLKL). The CM5 chip was coated with endotoxin-free Wt-hMLKL and Wt-mMLKL. The *K*_D_ values of Re-APO and Ox-APO for the Wt-hMLKL were 2.44 × 10^−7^ M and 4.998 × 10^−9^ M, and for the Wt-mMLKL were 2.295 × 10^−8^ M and 3.807 × 10^−10^ M, respectively (Fig. 3A, B). These results show that Re- and Ox-APO strongly bind to human and mouse MLKL, and Ox-APO showed ~50- and 165-fold stronger binding than Re-APO, respectively. We further examined the binding of APO to the Nt-hMLKL and Ct-hMLKL. Ox-APO exhibited strong binding to Nt-hMLKL (1.312 × 10^−6^ M), whereas Re-APO showed substantially weaker binding (1.14 × 10^−3^ M), representing roughly a 1000-fold difference (Fig. 3C). Similar to hMLKL, Nt-mMLKL bound strongly to Ox-APO (1.379 × 10⁻⁸ M) (Fig. 3D). However, no detectable binding was observed between Ox- or Re-APO and Ct-hMLKL (Fig. 3E). Together, these results demonstrate that Ox-APO binds more strongly to Wt-hMLKL and -mMLKL than Re-APO, particularly through interaction with the N-terminal domain.

### Modeling of Ox-APO binding to MLKL

**Fig. 4:**
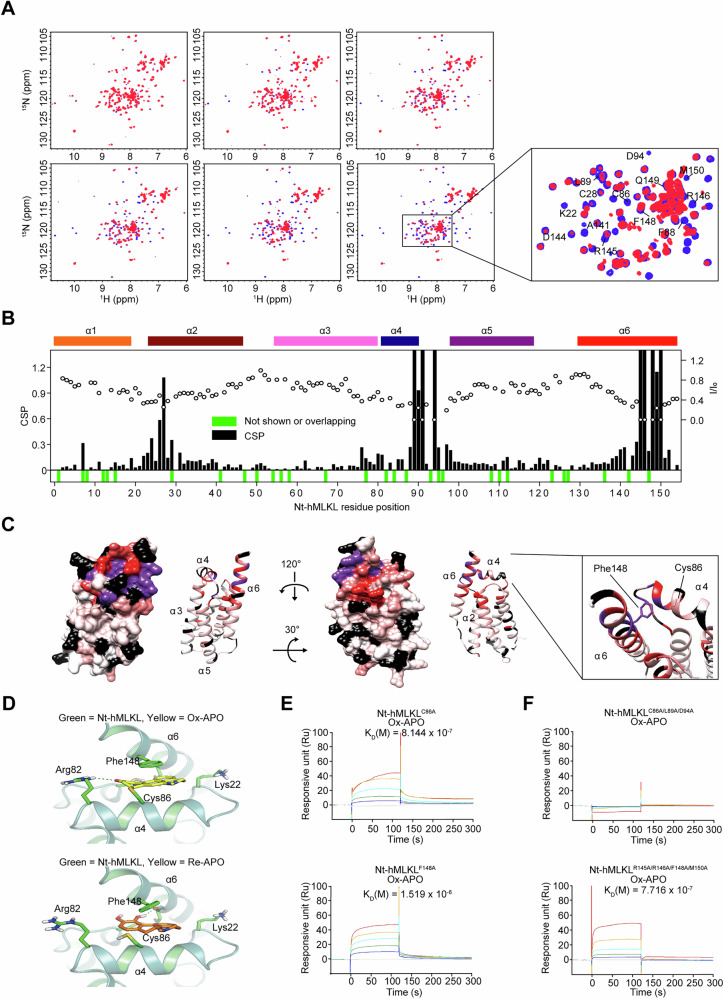
Structural analysis of the interaction between APO and Nt-hMLKL. **A** NMR analysis of Ox-APO binding to Nt-hMLKL. APO was pre-incubated for 0, 2, 4, 8, 12, and 24 h at 25 °C for auto-oxidation, and the NMR ^1^H-^15^N HSQC spectrum of Nt-hMLKL in the presence of APO (red) is superimposed on that of unbound Nt-hMLKL (blue). The labeled amino acid residues indicate an increase in CSPs due to their interaction with Ox-APO. **B** CSPs (histogram, left *y*-axis) and intensity ratio values (dot, right *y*-axis) of backbone amides of Nt-hMLKL in response to Ox-APO are plotted. α1 to α6 indicate the helices of Nt-hMLKL. *I*/*I*_0_ the intensity ratio, *I* the intensity of Nt-hMLKL with APO, *I*_0_ the intensity of unliganded Nt-hMLKL; green bar, amino acid residues overlapped by CSP peaks that are completely or barely visible. **C** CSP mapping onto the 3D conformer (PDB ID code 6ZPR). The UCSF Chimera program displays conformers in 3D as ribbon or sphere surfaces and colors amino acid residues from purple (highest) to white (lowest) based on the height of the CSP peaks. Residues not visible by NMR are colored black. **D** Comparison of the APO binding site of Nt-hMLKL (green; PDB ID code 6ZPR). Ox-APO (yellow) is shown as sticks. Key interacting residues are shown as sticks (covalent bond between Cys86 and Ox-APO; π–π stacking between Phe148 and Ox-APO core). **E**, **F** SPR analysis. Nt-hMLKL^C86A^ and Nt-hMLKL^F148A^ (**E**), and Nt-hMLKL^C86A/L89A/D94A^ and Nt-hMLKL^R145A/R146A/F148A/M150A^ (**F**) were immobilized on CM5 chips, and Ox-APO at concentrations of 0, 12.5, 25, 50, 100, and 200 μg/mL was flowed through to observe binding.

NMR analysis was performed to identify the specific binding sites. Two-dimensional ^1^H-^15^N heteronuclear single quantum correlation (HSQC) spectra were performed with Nt-hMLKL according to the oxidation state of APO, which was incubated in an aqueous solution for various times for auto-oxidation. The resonance intensity increased with the degree of APO oxidation (Fig. 4A). The superimposed spectra showed that the resonance intensity of Ox-APO was stronger than that of Re-APO at 24 h (Fig. 4A). Some resonances in Nt-hMLKL showed chemical shift perturbations (CSPs) in the presence of Ox-APO, suggesting that Ox-APO binds more strongly to Nt-hMLKL than Re-APO. Nt-hMLKL consists of six α-helices (designated α1–α6). Su et al. showed that the α1, α2, α3, and α5 combine to form a membrane insertion bundle, and α6 interacts with the interface between the α2 and α5, while the shorter α4 helix is perpendicularly oriented at the top of the four-helix bundle [46]. The HSQC peaks of Nt-hMLKL were assigned to trace the residues involved in Ox-APO binding (Fig. 4B). Significant CSPs were observed in the α4 and α6 helices, particularly at residues Cys86, Leu89, Asp94 in α4, Arg145, Arg146, Phe148, and Met150 in α6, suggesting that these residues are critical for Ox-APO binding. Ox-APO-induced CSPs were then mapped onto the 3D structure of Nt-hMLKL (Fig. 4C). Based on the CSP data, the molecular docking studies were performed to predict the binding modes of Re-APO and Ox-APO on Nt-hMLKL (Fig. 4D). The docking results showed that Ox-APO binds more strongly to Nt-hMLKL than Re-APO. This enhanced binding was attributed to a covalent bond formed with Cys86 and a π–π interaction with Phe148, which were not observed for Re-APO (Fig. 4D). Notably, several key peripheral residues involved in APO binding to human MLKL, such as Cys86, are not conserved in mMLKL, which is consistent with the known species-specificity of compounds like NSA. However, our 3D docking model of APO with mMLKL suggests that APO can interact with Lys81—along with Ser79, Arg105, and Asp106—residues known to participate in mMLKL oligomerization (Fig. S3) [5]. These findings suggest that APO may still inhibit mMLKL oligomerization through alternative interaction residues, even in the absence of conserved cysteines like Cys86.

NSA is an MLKL inhibitor known to covalently target Cys86 [4]. In the SPR competition assay alongside Ox-APO for Wt-hMLKL, NSA competed with Ox-APO for binding to hMLKL (Fig. S4). However, single mutations at Cys86 and Phe148 in Nt-hMLKL showed binding to Ox-APO but ~10-fold weaker binding (Fig. 4E), suggesting that both residues are required for the strong binding. When we performed SPR analysis using a triple mutant in the α4 helix of Nt-hMLKL, Nt-hMLKL^C86A/L89A/D94A^, Ox-APO showed no binding (Fig. 4F). In addition, a quadruple mutant containing R145A, R146A, F148A, and M150A in α6 of hMLKL, hMLKL^R145A/R146A/F148A/M150A^, retained binding to Ox-APO (Fig. 4F). All these results suggest that C86, L89, D94, and Phe148 of hMLKL are the important residues for OX-APO binding.

### Ox-APO effectively ameliorates DSS-induced colitis and APAP-induced liver injury

**Fig. 5:**
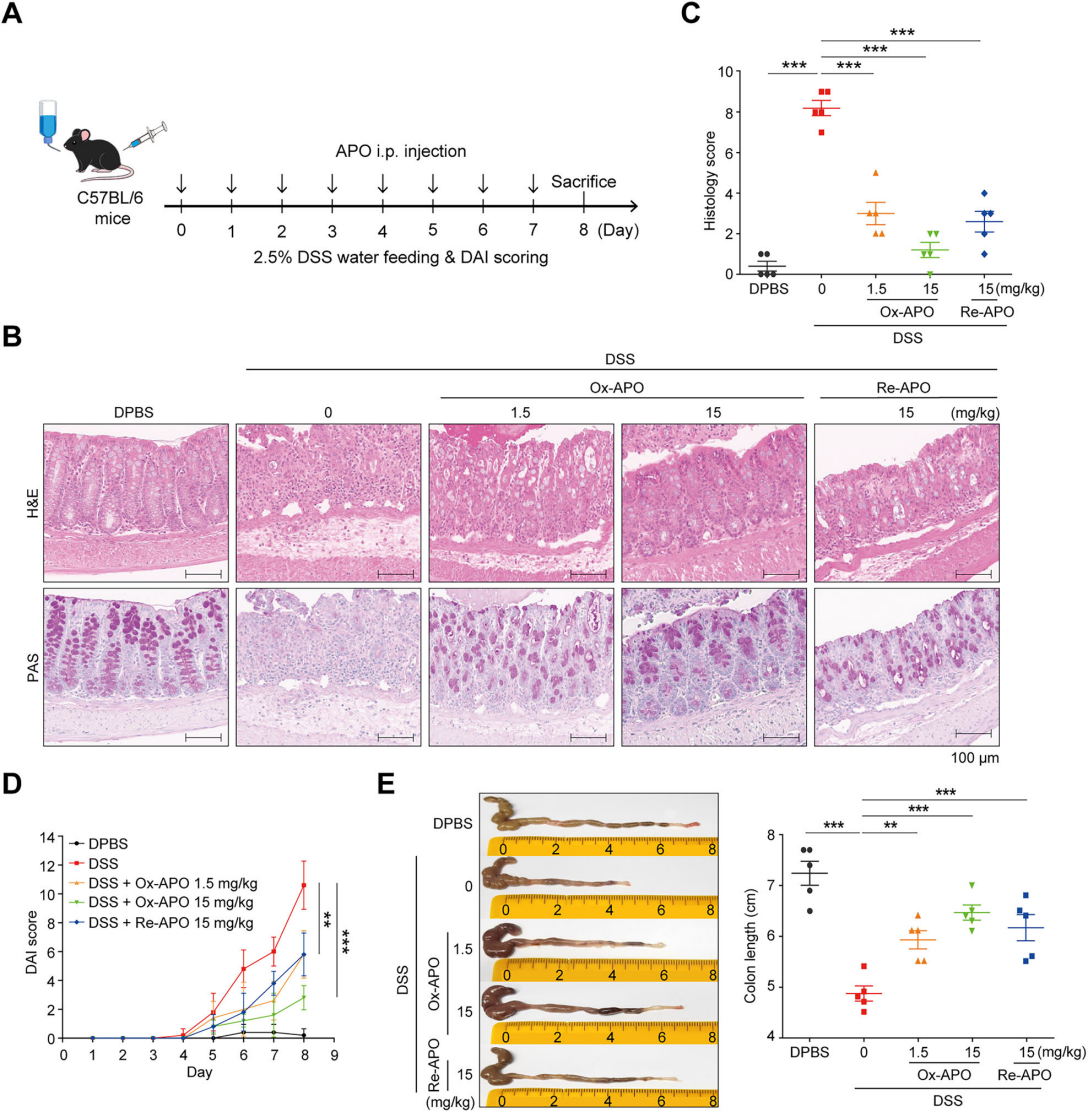
APO ameliorates IBD in the DSS-induced mouse colitis model. **A** Experimental design of DSS-induced colitis in mice. C57BL/6 mice were given a 2.5% DSS in the drinking water for 8 days. Re-APO or Ox-APO was administered intraperitoneally daily for 7 days starting on day 0. On day 8, the mice were sacrificed, and the colon tissues were collected. **B** Mouse colon tissues were collected, and the effect of Re-APO or Ox-APO on histopathology was evaluated by H&E and PAS staining. **C** Histological scores of each group were evaluated. Mean ± SD (*n* = 5). *p < 0.05, ***p < 0.001, one-way ANOVA. **D** DAI of colitis was measured as mean ± SD (*n* = 5). **p < 0.01, ***p < 0.001, *t*-test. **E** Colon length was measured. Representative images of the colon (left) and colon length (right). Mean ± SD (*n* = 5). *p < 0.05, **p < 0.01, ***p < 0.001, one-way ANOVA.

Based on the results of the in vitro mouse cell experiments and SPR analysis with mMLKL (Figs. 2E and 3B), we first performed an in vivo experiment using a mouse DSS-induced colitis model to observe the inhibitory effect of APO on necroptosis-related colitis symptoms. C57BL/6 mice were treated with 2.5% DSS in drinking water for 8 days, and the mice were intraperitoneally (i.p.) administered APO daily (Fig. 5A). Body weight and disease activity index (DAI) scores were recorded daily, and colon tissues were collected at the end of the experiment. When DSS mice were treated with DPBS, they exhibited typical IBD symptoms, including damage to crypt architecture, inflammatory cell infiltration, submucosal edema, and impaired mucin secretion in the colon. These phenomena were significantly alleviated by increasing the dose to 0.15, 1.5, or 15 mg/kg APO, the oxidative state of which was not determined, and pathological changes in tissue sections of H&E and PAS staining were improved (Fig. S5A–D). Next, we produced both Ox-APO and Re-APO to compare the effects in the same DSS colitis model. Ox-APO resulted in better histopathologic scores in H&E and PAS staining than Re-APO at the same 15 mg/kg (Fig. 5B). The average histologic score for Ox-APO at 1.5 mg/kg was 3.0, which was similar to the score of Re-APO (2.6) at 15 mg/kg, and Ox-APO at 15 mg/kg was a better improvement of 1.2 score (Fig. 5C). The DAI score and colon length also showed that Ox-APO was more effective than Re-APO (Fig. 5D, E).

**Fig. 6:**
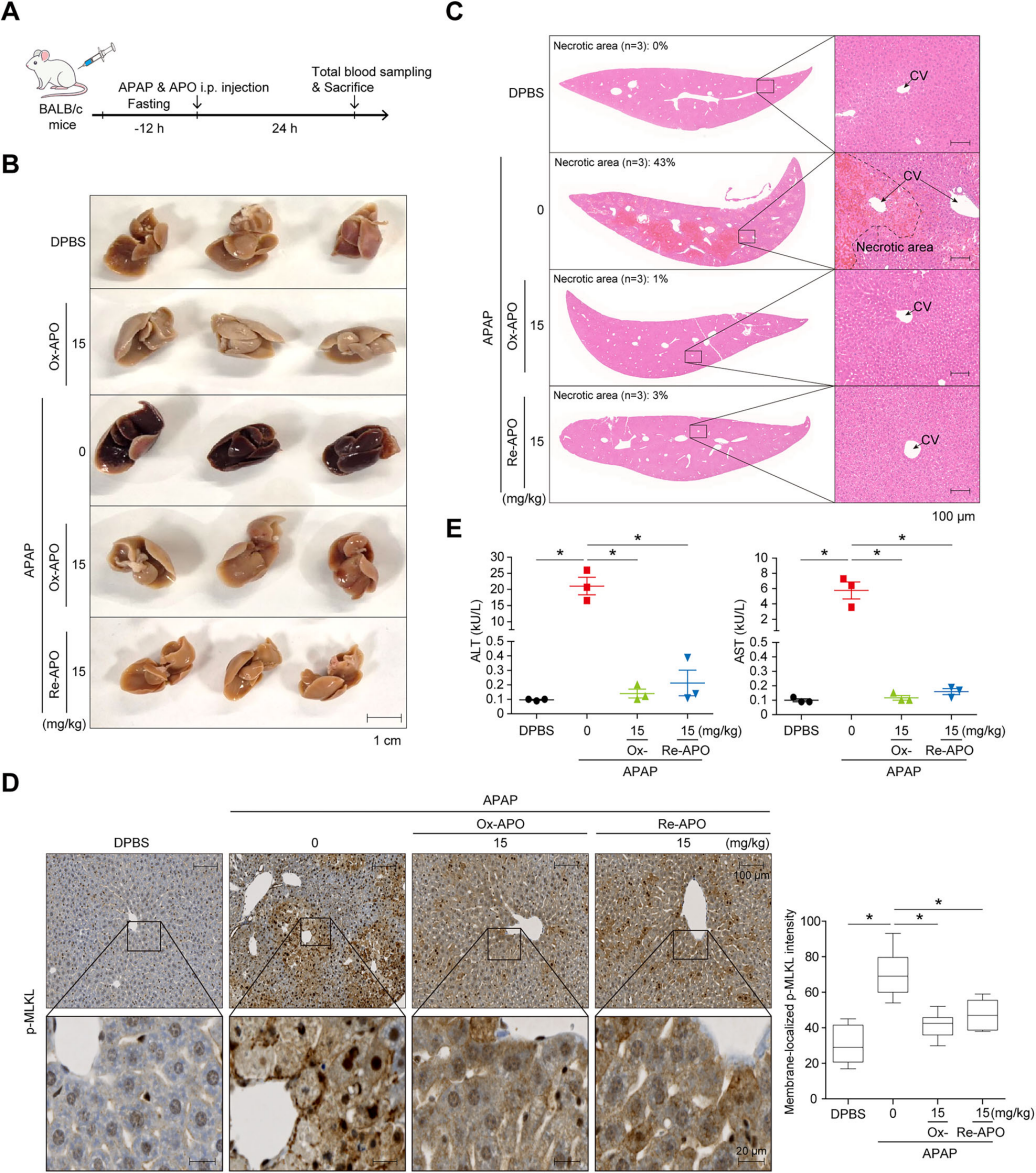
APO protects against liver injury in an APAP-induced mouse model. **A** BALB/c male mice (6–8-weeks-old) received an i.p. injection of 400–500 mg/kg APAP in the presence or absence of APO after a 12 h fasting period. Livers were perfused and harvested 24 h after the injection. **B** Gross morphology of liver tissues (*n* = 3 per group) after 500 mg/kg APAP injection to induce severe liver injury. **C** H&E staining of liver tissues after APAP injection. The necrotic area was quantified using ImageJ by measuring the unstained regions and calculating their proportion relative to the total tissue area. The boxed images are magnifications. **D** Membrane-localized p-MLKL was assessed by IHC staining of paraffin-embedded liver sections. The boxed images are magnifications. Quantification of membrane-localized p-MLKL signal intensity was performed on over 100 cells per group using Zen imaging software (Zeiss), with normalization to cytosolic p-MLKL intensity. Data are presented as mean ± SD. *p < 0.001; one-way ANOVA. **E** Serum AST and ALT levels were evaluated to assess liver injury after APAP injection. Mean ± SD (*n* = 3). *p < 0.001, ns not significant, one-way ANOVA.

We used another necroptosis-related disease model of APAP-injected liver injury to confirm the in vivo effect [47]. BALB/c mice were fasted for 12 h and then i.p. injected with a 400-500 mg/kg APAP and then injected once with 15 mg/kg Ox-APO or Re-APO for 24 h (Fig. 6A). The liver tissues were dark brown when APAP was injected, but these colors of Ox-APO treated group and Re-APO treated group at 15 mg/kg were almost similar to the normal mouse group treated with DPBS alone (Fig. 6B). Histological analysis of liver tissues by H&E staining revealed extensive necrotic areas, covering approximately 43% of the tissue and characterized by hepatocyte death in the APAP-treated group (Fig. 6C). In contrast, hardly any necrotic areas were observed in the Ox-APO and Re-APO treated groups. And the immunohistochemical analysis (IHC) showed a significant increase in membrane p-MLKL post APAP treatment, but a dramatic decrease in the Ox-APO– and Re-APO–treated groups (Fig. 6D). Serum alanine aminotransferase (ALT) and aspartate aminotransferase (AST) levels were significantly improved by Ox-APO and Re-APO treatments, almost to the baseline level of the DPBS-treated group (Fig. 6E).

And we performed experiments using C57BL/6 MLKL^−^^/^^−^ mice to determine whether Ox-APO inhibits alternative mechanisms beyond necroptosis inhibition. We used the same APAP-induced liver injury protocol (Fig. S6A). The gross morphology of livers showed less hemorrhaging (Fig. S6B). The H&E staining of liver tissues, serum ALT and AST levels, and TUNEL-positive (apoptosis) and 4-HNE-positive (ferroptosis) IHCs showed hepatic damage by alternative mechanisms of apoptosis and ferroptosis following APAP injection (Fig. S6C–E). However, all of these findings were significantly reduced by treatment with 15 mg/kg Ox-APO. Recent studies have shown that APO reduces ROS production, thereby suppressing apoptosis [48], and downregulates the expression of proteins involved in lipid peroxidation, contributing to the inhibition of ferroptosis [49]. All these demonstrate that Ox-APO can suppress necroptosis and multiple forms of regulated cell death, highlighting its broader therapeutic potential.

## Discussion

MLKL is a key mediator in the necroptosis pathway, consisting of an N-terminal four-helical bundle domain and a C-terminal pseudokinase domain connected by two brace helices [50]. The N-terminal domain is typically inactive due to the autoinhibitory first brace helix (α6) [51]. Upon activation by RIP1 and RIP3, which phosphorylate the C-terminal pseudokinase domain, the autoinhibitory helix unfolds, allowing the N-terminal domain to form multimers that integrate into the cell membrane, ultimately leading to membrane rupture and necroptosis [4]. Necroptosis has been implicated in many human inflammatory diseases, including acute pancreatitis, multiple sclerosis, liver injury, IBD, and allergic colitis. Therefore, specifically targeting MLKL to prevent necroptosis is an attractive strategy for drug discovery because it inhibits the release of DAMP molecules and spares the RIP1- and RIP3-related functions. Currently, two types of MLKL inhibitors, NSA and xanthine class inhibitors, which covalently target Cys86, have been reported [4, 51]. NSA targets human MLKL but not the mouse form, which limits its application. Here, we discovered APO as a promising potential inhibitor with a different mechanism of action that prevents MLKL oligomerization, thereby preventing necroptosis.

APO, a derivative of morphine, is formed by acid-catalyzed rearrangement. As an FDA-approved drug, APO has been used as a non-selective, direct-acting dopamine receptor agonist to reverse the “off” episode in patients with late-stage Parkinson’s disease [52]. It is also used as an emesis inducer for antiemetic screening [53]. In this study, we identified a novel function of APO, specifically its oxidized form, Ox-APO, which inhibits necroptosis by blocking MLKL oligomerization and its migration to the cell membrane to form necrotic pores independently of RIP1 and RIP3. Western blotting and confocal microscopy revealed that even Ox-APO showed little inhibition of MLKL phosphorylation upon TBZ stimulation, but it significantly inhibited MLKL tetramerization and octamerization. SPR and NMR studies confirmed that Ox-APO binds strongly to both Wt-hMLKL and Nt-hMLKL. The fact that Ox-APO shows no binding to Nt-hMLKL^C86A/L89A/D94A^ but binds strongly to Nt-hMLKL^R145A/R146A/F148A/M150A^ suggests that Ox-APO interacts mainly with the α4 domain of Nt-hMLKL via covalent, ionic, and hydrophobic interactions involving at least three residues: Cys86, Leu89, and Asp94. These residues form a cavity on top of the four-helix bundle composed of α1, α2, α3, and α5, adjacent to Cys86 and Phe148. Notably, Cys86 is the target residue for the NSA, which covalently binds to MLKL and prevents its oligomerization [4]. Phe148 stabilizes the autoinhibitory α6 and four-helix bundle via the π–π interaction, and xanthine class inhibitors (BI-8925 and TC13172) stabilize the Phe148 of the autoinhibitory α6 and four-helix bundle [51]. Notably, despite the absence of a Cys86-equivalent residue in mouse MLKL, our results suggest that Ox-APO may interfere with oligomerization by interacting with Lys81, a residue previously implicated in mMLKL oligomerization [5]. Further structural analyses are planned to precisely characterize the molecular basis of this interaction. Collectively, these findings highlight that Ox-APO has unique binding characteristics distinct from NSA and is effective against both human and mouse MLKL.

When two disease models represent necroptosis, Ox-APO treatment effectively alleviated DSS-induced IBD and APAP-induced liver injury in mice. APO can be auto-oxidized in solution over time in the presence of light [43]. Ox- and Re-APO under strict conditions showed that Ox-APO has a stronger clinical effect. Re-APO was also effective in alleviating DSS-induced colitis symptoms, but about ten times weaker than Ox-APO at the same concentration.

In the APAP-induced liver injury model, both Ox-APO and Re-APO reduced liver damage. This is likely attributable to APO’s ability to inhibit multiple forms of regulated cell death beyond necroptosis [48, 49]. Studies using MLKL^–/–^ mice confirmed that necroptosis plays a significant role in APAP-induced hepatic injury. Notably, APO treatment mitigated liver damage not only by targeting necroptosis but also by modulating apoptosis and ferroptosis. These results are consistent with previous reports indicating that APO can influence multiple regulated cell death pathways, supporting its potential as a multi-modal cell death regulator and a promising candidate for therapeutic development. While oxidation enhances APO’s efficacy as an MLKL inhibitor, excessive oxidation reduces compound stability, causes precipitation, and alters pharmacokinetics [52]. In PD formulations used for Parkinson’s disease, pH adjustment or the use of antioxidants is required to protect APO from auto-oxidation in solution. However, optimal oxidation of APO could reduce the dose required to treat necroptosis, thereby reducing potential side effects. Therefore, optimization of the oxidation state to balance appropriate pharmacokinetic properties is necessary for its application in necroptosis-related diseases. Although APO induces an emetic response, the effect of Ox-APO on emesis needs to be further investigated because the lack of specific neuromuscular components in the brainstem prevents rodents from vomiting [54]. Further application studies of Ox-APO as a necroptosis inhibitor are needed, for example, in cell or organ preservation under poor environmental or ischemic conditions during transfer for cell therapy and organ transplantation. In conclusion, this study identifies APO, particularly Ox-APO, as a novel potential necroptosis inhibitor through drug repurposing. Ox-APO strongly binds to both human and mouse MLKL and inhibits necroptosis by targeting the formation of cell membrane pores. Thus, Ox-APO is expected to serve as a promising drug candidate for the treatment of necroptosis-related diseases.

## Experimental model and study participant details

### Chemical library

A clinical chemical library containing 2150 items (Korea Chemical Bank, South Korea), under phase 1–3 clinical trials, including FDA-approved drugs, was used to screen for necroptosis inhibitors. Each chemical was dissolved in DMSO and diluted to 20 μM in DPBS for treatment.

### Cell culture and reagents

THP-1-HMGB1-Lucia^TM^ cells (thp-gb1lc, InvivoGen, San Diego, CA, USA) stably expressing HMGB1-luciferase were maintained in a complete medium supplemented with 25 mM HEPES, 100 μg/mL Zeocin (ant-z-1, InvivoGen), and Mycozap (VZA-2031, Lonza, Swiss). THP-1 cells (TIB-202, ATCC, Manassas, VA, USA) were treated with a complete medium containing 500 nM phorbol 12-myristate 13-acetate (PMA) for 3 h for cell maturation and adherence. HEK293T cells and the mouse monocytic cell line J774A.1 (TIB-67, ATCC) were cultured in complete medium, respectively. All cells were cultured in an incubator at 37 °C with 5% CO_2_.

### Mouse study of DSS-induced colitis and APAP-induced liver injury

For DSS-induced colitis, male C57BL/6 mice aged 6–8 weeks (Central Lab Animal Inc., S. Korea) were administered 2.5% DSS (36–50 kDa, 9011-18-1, MP Biomedicals, Solon, OH, USA) in their drinking water for 8 days. The 2.5% DSS-supplemented drinking water was prepared by dissolving 12.5 g of DSS powder in 500 mL of distilled water and was freshly prepared and replaced daily throughout the 8 days. Body weight and DAI score were recorded daily. The DAI score was calculated based on weight loss (no weight loss, 1–5%, 5–10%, 10–15%, and >15% weight loss, respectively, representing DAI scores of 0, 1, 2, 3, and 4), stool consistency/diarrhea (normal = 0, loose stools = 2, and watery diarrhea = 4), and bleeding (no bleeding = 0, slight bleeding = 2, gross bleeding = 4). At the end of the experiment, the mice were sacrificed, and tissue samples were collected.

For APAP-induced liver injury, male BALB/c mice aged 6–8 weeks (Central Lab Animal Inc., S. Korea) and male MLKL^–/–^ C57BL/6 mice aged 6–8 weeks (Cyagen, Santa Clara, CA, USA) were administered acetaminophen (APAP; 400–500 mg/kg, 103-90-2, Sigma-Aldrich) by i.p. injection to induce liver injury in the presence or absence of APO after 12 h of fasting. After 24 h, the mice were sacrificed to obtain liver tissues for further analysis.

All animal experiments were performed with random assignment to treatment groups and with blinding of investigators during data collection and analysis.

## Method details

### Induction of necroptosis and luciferase assay

THP-1-HMGB1-Lucia^TM^ cells were pretreated with the pan-caspase inhibitor Z-VAD-FMK (50 μM, tlrl-vad, InvivoGen) and each chemical for 1 h, and followed by TNF-α (100 ng/mL, TNF0501, NKMAXBio, Seongnam, S. Korea) and Smac mimetic BV6 (10 μM, B1332-5, Biovision, Santa Clara, CA, USA), termed TBZ mixture, were added for necroptosis screening. The RIP1 inhibitor, NEC-1 (1846, Biovision), and MLKL inhibitor, NSA (432531-71-0, Sigma-Aldrich, St. Louis, MO, USA), were used as controls. The cell supernatant was harvested to measure HMGB1 levels using a luciferase assay kit (E1500, Promega, Madison, WI, USA) by a luminometer (Centro XS3 LB 960, Berthold Technologies, Bad Wildbad, Germany). The inhibition of necroptosis (%) was calculated as: (sample-treated data – medium-only data) × 100/(TBZ-treated data – medium-only data). APO [STK088477, (6aS)-6-methyl-5,6,6a,7-tetrahydro-4H-dibenzo[de,g]quinoline-10,11-diol, Vitas-M Laboratory, Hong Kong] was stored at −70 °C after aliquoting with DMSO. APO was used after dilution in DPBS. APO is susceptible to auto-oxidation in solution in the presence of light and air [43]. DPBS containing 5 mM DTT was used for Re-APO. Ox-APO was prepared by incubating APO in DPBS at 25 °C for 24 h in the presence of light and air, followed by removal of any precipitate by filtration through 0.2 μm [55].

### PI uptake assay

PI uptake assays were performed in triplicate to evaluate pore formation on the plasma membrane. THP‑1 cells (2 × 10⁵ cells/well) were seeded in black-walled, clear-bottom 96-well plates and treated with TBZ (TNF, BV6, and z-VAD-FMK) in the presence or absence of candidate compounds for 8 h to induce necroptosis. For the chemically induced MLKL-gyrase dimerization experiments, J774A.1 cells (2 × 10⁵ cells/well) were seeded and treated with Dox and coumermycin for 4 h, followed by Ox-APO treatment overnight. PI (556463, BD Biosciences, San Jose, CA, USA) was added at a final concentration of 1 μg/mL for 10 min before measurement. Fluorescence was recorded using a Varioskan Flash 3001 plate reader (Thermo Fisher Scientific, Waltham, MA, USA).

### Western blot analysis

The cells were harvested and lysed in 1× RIPA buffer (R4100-010, GenDEPOT, Katy, TX, USA) containing protease (P3100-001, GenDEPOT) and phosphatase (1862495, Thermo Fisher Scientific) inhibitors. WCLs were then centrifuged at 20,000×*g* for 30 min at 4 °C and subjected to non-reducing or reducing 8–12% SDS-PAGE gel electrophoresis. The membrane was blocked with 5% non-fat milk in TBS and probed with antibodies. Antibodies to hMLKL (14993S), mMLKL (97705S), phospho-hMLKL (Ser358) (91689S), phospho-mMLKL (Ser345) (37333S), phospho-hRIP1 (Ser166) (65746S), mRIP1 (3493S), phospho-mRIP1 (Ser321) (38662S), phospho-hRIP3 (Ser227) (93654), mRIP3 (95702S), p-mRIP3 (Thr231/Ser232) (91702S), and LDH (2012S) (Cell Signaling Technology, Danvers, MA, USA), and GAPDH (LF-PA0018, Abfrontier, S. Korea), hRIP1 (ab72139, Abcam, Cambridge, UK), hRIP3 (sc-135170, Santa Cruz Biotechnology, Dallas, TX, USA), HMGB1 (ab78923, Abcam), and LAMP1 (ab24170, Abcam) antibodies were used.

### Measurement of released HMGB1 levels

TBZ-treated cell supernatants were harvested and centrifuged using Amicon ultra centrifugal filters 10 kDa (UFC501096, Millipore, Billerica, MA, USA) at 4000×*g* for 1 h. The concentrated supernatants were analyzed using SDS-PAGE to assess released HMGB1 levels.

### Cell membrane fractionation

Cell membrane fractionation was performed to determine whether MLKL oligomers were retained in the cell membrane fraction [31]. Briefly, THP-1 cells were harvested and resuspended in 20 mM TBS (pH 7.4) containing 10 mM KCl, 1 mM MgCl_2_, protease inhibitor (P3100-001, GenDEPOT), and phosphatase inhibitor (1862495, Thermo Fisher Scientific). The cell suspension was passed through a 22-gauge needle and centrifuged at 20,000×*g* for 15 min and stored as the cytoplasmic fraction. The pellet was resuspended in lysis buffer and stored as the crude membrane fraction.

### Confocal microscopy

THP-1 cells were washed and fixed in 4% paraformaldehyde and incubated in 0.25% Triton X-100 for 10 min, blocked for 30 min with 5% BSA-PBS, and stained with the MLKL and CD36 (ab252922, Abcam) antibody. The nuclei were stained with DAPI. Confocal microscopy (FV1000, Olympus, Tokyo, Japan) was used for image analysis. The signal intensity was measured using FluoView FV1000 software.

### DNA constructs and recombinant proteins

The hMLKL plasmid (RC213152, OriGene, Rockville, MD, USA), and recombinant Wt-hMLKL (ab241453, Abcam) and Wt-mMLKL (MBS1379843, MyBioSource, San Diego, CA, USA) proteins were purchased. Nt-hMLKL (2–154), Nt-mMLKL (1–180), and Ct-hMLKL (183–471) were generated using the pBT7-N-His vector. Nt-hMLKL^C86A^, Nt-hMLKL^F148A^, -hMLKL^C86A/L89A/D94A^, and Nt-hMLKL^R145A/R146A/F148A/M150A^ were generated using the Site-directed Mutagenesis Kit (EZ004S, Enzynomics, S. Korea). These constructs were subcloned into pBT7-N-His and pET-15b plasmid vectors. *E. coli* BL21 cells transformed with the cloned expression vectors were cultured in LB medium and supplemented with 100 μg/mL ampicillin until an OD at 600 nm of 0.5–0.6 was reached. The cultures were induced by 1 mM IPTG (367-93-1, Sigma-Aldrich). The cells were lysed and centrifuged. The clear supernatants were subjected to Ni-NTA affinity chromatography (175018169, QIAGEN, Hilden, Germany). Plasmids encoding mMLKL (1–464)–gyrase fusion constructs were kindly provided by Prof. James Murphy (Cell Signalling and Cell Death Division, The Walter and Eliza Hall Institute of Medical Research, Parkville, Victoria 3052, Australia) [44].

### SPR analysis

The interactions of Wt-hMLKL, Wt-mMLKL, Nt-mMLKL, Nt, and Ct-hMLKL proteins with Re-APO or Ox-APO were detected through SPR analysis using Biacore™ T200 (Cytiva, Marlborough, MA, USA). Each MLKL protein was immobilized on a CM5 sensor chip. To derive the R_ligand_ values, coupling processes were performed based on the molecular weights of the MLKL protein (MW ligand) and Re-APO or Ox-APO (MW analyte). Re-APO or Ox-APO was injected into an HBS buffer (10 mM HEPES, 150 mM NaCl, 3.4 mM EDTA, and 0.005% Tween 20, pH 7.4) at 25 °C (10 μL/min flow rate). Sensorgrams were recorded and analyzed in real-time using the control software in the BIAcore T200 system. All data were analyzed using the BIA evaluation software (Cytiva).

### NMR experiments

To obtain ^13^C/^15^N-labeled Nt-hMLKL protein, the transformed *E. coli* cells were grown in an M9 minimal medium containing ^15^N-NH_4_Cl and ^13^C-glucose (CIL, Tewksbury, MA, USA). All labeled materials were purchased from Cambridge Isotope Laboratories. The cells were stimulated for protein expression with IPTG. The Nt-hMLKL protein was purified using Ni-affinity column chromatography, followed by TEV cleavage during dialysis. Further purification was performed via size-exclusion column chromatography using the Superdex-75 column (Cytiva). The concentration of the Nt-hMLKL protein was determined by UV absorbance at 280 nm.

NMR experiments were conducted in NMR buffer (pH 7.0, 20 mM HEPES, 100 mM NaCl, 5% D_2_O) at 25 °C. The backbone CSs of Nt-hMLKL were assigned using triple resonance NMR spectra (HNCACB/HNcoCACB and HNCO/HNcaCO). The CS values of Nt-hMLKL (BMRB code: 25135) were used as a reference. The NMR data were processed using the NMRpipe program and analyzed using the NMRFAM-Sparky program [56, 57]. The CSP experiments were performed to monitor drug molecule binding, and the differences in CSs were calculated using a previously reported method [58]. The ^1^H-^15^N HSQC spectra were recorded in the absence and presence of APO. The Ox-APO concentration was estimated from peak integrals in the 1D spectrum. The CSP data were visualized on the structure of Nt-hMLKL using the UCSF Chimera program (https://www.cgl.ucsf.edu/chimera/).

### Tissue sample preparation and histologic evaluation

Each mouse colon was removed, measured in length, cut open, fixed in Bouin’s fixative (50% ethanol/5% acetic acid in distilled water), and embedded in paraffin. Tissue sections were stained with H&E (Sigma-Aldrich). Histological evaluation of the colitis lesions along the entire colon was performed using the scoring system: degree of crypt structural damage (0–3), degree of inflammatory cell infiltration (0–3), and degree of submucosal edema (0–3) [59]. For PAS staining, deparaffinized and rehydrated tissue sections were treated with 0.5% periodic acid (10450-60-9, Junsei Chemical Co., Tokyo, Japan) and stained with Schiff’s reagent (3952016, Sigma-Aldrich). Sections were dehydrated in ethyl alcohol and coverslipped.

For liver tissue preparation, each liver was perfused with DPBS and then fixed in 4% paraformaldehyde (PFA; PC2031-050-00, Biosesang, S. Korea) before embedding in paraffin. Sections were stained with H&E, and necrotic areas were quantified using ImageJ by calculating the proportion of pale or unstained regions relative to the total tissue area. Mouse liver sections were subjected to IHC staining to assess distinct forms of cell death: an anti-p-MLKL antibody (MA5-32752, Thermo Fisher Scientific) to evaluate p-MLKL localization (necroptosis), a TUNEL assay kit (4810-30-K, R&D Systems) for apoptosis, and an anti-4-HNE antibody (GTX01087, GeneTex) for ferroptosis.

### Quantification and statistical analysis

Data analysis was performed using the Student’s *t*-test or one-way analysis of variance (ANOVA) test using GraphPad Prism software (GraphPad Software, Inc., San Diego, CA, USA). Results were presented as the mean and standard error of the mean (SEM) or standard deviation (SD), as indicated in the figure legends. Statistical significance was set at a minimum of *p* < 0.05.

## Supplementary information

Figs. S1–S6 and Tables S1

Unedited blot and gel images

## Data Availability

Data supporting the findings of this study are available from the corresponding author upon reasonable request.
