# Supplementary material for: Apomorphine is a novel necroptosis inhibitor targeting mixed lineage kinase domain-like protein oligomerization
Source: Cell Death Discov. 2025 Oct 13;11:457. doi: 10.1038/s41420-025-02763-8 (PMC12518639; doi:10.1038/s41420-025-02763-8)
Supplement: Supplementary file 1 — Figs. S1–S6 and Tables S1 [file 41420_2025_2763_MOESM1_ESM.docx]

**Apomorphine is a novel necroptosis inhibitor targeting mixed lineage kinase domain-like protein oligomerization**

**Myeonggil Han^1,2^, Dong-Hyun Seo^6,7^, Man Sup Kwak^1,3^, In Ho Park^3,5^, Woo Joong Rhee^1,3^, Hee Sue Kim^1^, Eunkyeong Jeon^1^, Je-Jung Lee^1,3^, Cheol Ho Park****^1^, Nam Doo Kim^8^, Taebo Sim^2,5^, You-Sun Kim^9,10^, Kyoung-Seok Ryu^6,7,**^,** **Jeon-Soo Shin^1-4,*^**

^1^Department of Microbiology, Yonsei University College of Medicine, Seoul, South Korea.

^2^Brain Korea 21 FOUR Project for Medical Science, Yonsei University College of Medicine, Seoul, South Korea.

^3^Institute for Immunology and Immunological Diseases, Yonsei University College of Medicine, Seoul, South Korea.

^4^Center for Nanomedicine, Institute for Basic Science (IBS), Yonsei University, Seoul 03722, South Korea.

^5^Department of Biomedical Sciences, Yonsei University College of Medicine, Seoul 03722, South Korea.

^6^Ochang Center, Korea Basic Science Institute, 162 Yeongudanji-Ro, Ochang-Eup, Cheongju-Si, Chungcheongbuk-Do 28119, South Korea.

^7^KBSI School of Bioscience, University of Science and Technology, Daejeon 34113, South Korea.

^8^Voronoibio Inc., 32 Songdogwahak-ro, Yeonsu-gu, Incheon, 21984, South Korea

^9^Department of Biochemistry, Ajou University School of Medicine, 164 Worldcup-ro, Yeongtong-gu, Suwon, 16499, Republic of Korea

^10^Department of Biomedical Sciences, Graduate School of Ajou University, 164 Worldcup-ro, Yeongtong-gu, Suwon, 16499, Republic of Korea

Correspondence should be addressed to jsshin6203@yuhs.ac*

**This file includes:**

**Figs. S1-S6 and Table. S1**

**Supplementary Figures and Figure Legends**

**
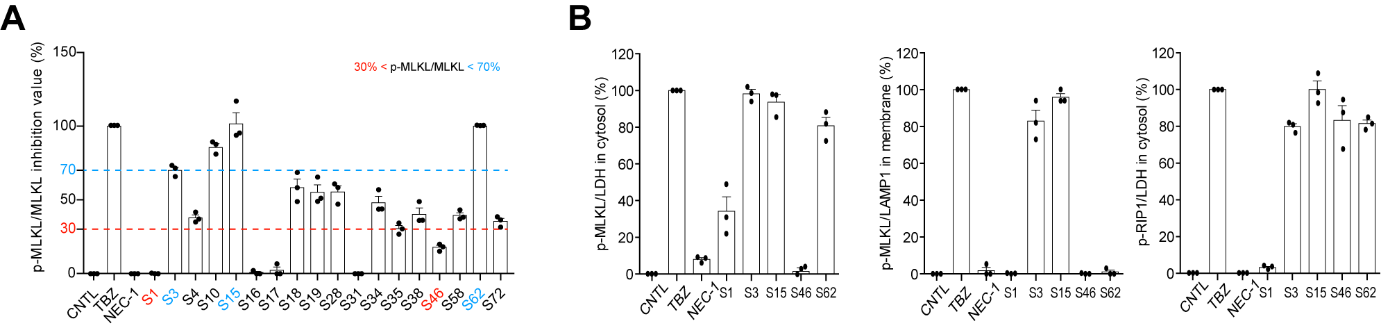
**

**Fig. S1 Assessment of p-MLKL inhibition to identify MLKL-targeting molecules.** (A) Quantification graph corresponding to Fig. 1C. THP-1 cells were treated with TBZ in the presence of secondarily selected compounds (S1–S72, 20 μM). Whole-cell lysates (WCLs) were collected and subjected to Western blot analysis under reducing conditions using antibodies against p-MLKL, MLKL, and GAPDH. The p-MLKL/MLKL ratio was quantified and compared to that of TBZ-treated cells. Data represent the mean of three independent experiments. (B) Quantification graph corresponding to Fig. 1D. THP-1 cells were treated with TBZ in the presence of third-round selected compounds (S1–S62, 20 μM). Cytosolic and membrane fractions were collected and analyzed by Western blot under reducing conditions. The levels of p-MLKL and p-RIP1 were normalized to LDH (cytosolic marker) and LAMP1 (membrane marker), respectively. Quantified ratios were compared to those of TBZ-treated cells (n = 3 independent experiments).

**
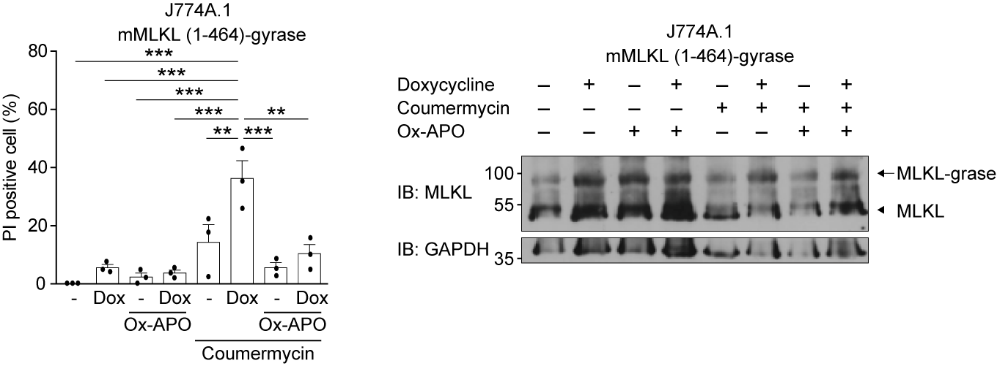
**

**Fig. S2 Ox-APO inhibits necroptosis induced by chemically induced MLKL dimerization.** J774A.1 cells were transfected with plasmids encoding mMLKL (1–464)-gyrase. mMLKL-gyrase expression was induced with 10 ng/mL doxycycline (Dox), followed by 700 nM coumermycin treatment for 4 h to promote dimerization. Then, Ox-APO (20 μM) was added, and the cells were incubated overnight. PI uptake was measured using a microplate reader, and the values were normalized to those of the untreated group. Data represent mean ± SEM (n = 3). ***p* < 0.01, ****p* < 0.001, one-way ANOVA. The expression of mMLKL-gyrase was confirmed by SDS-PAGE of WCLs, followed by immunoblotting with the indicated antibodies. The arrowhead indicates endogenous MLKL, while the tailed arrow denotes the mMLKL–gyrase fusion protein.

**
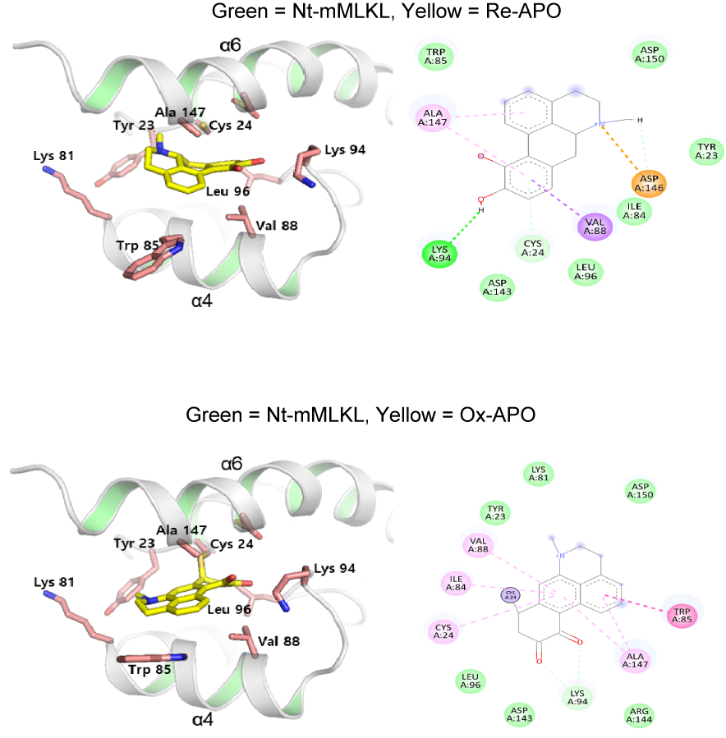
**

**Fig. S3 Docking model of APO binding to mMLKL.** Structural model of Ox-APO (yellow) binding to Nt-mMLKL (green). Key interacting residues are shown as sticks. Compared to Re-APO, Ox-APO forms additional interactions with several residues of Nt-mMLKL, including Lys81 (K81), which is known to be critical for mMLKL oligomerization.

**
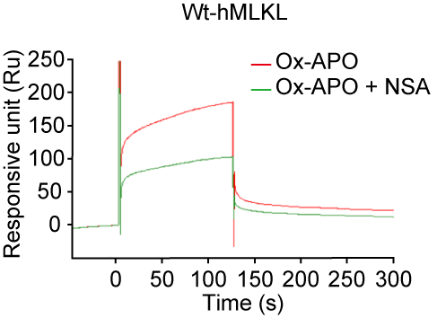
**

**Fig. S4 Competition assay of NSA with Ox-APO by SPR.** Wt-hMLKL was immobilized on a CM5 chip and 750 μM Ox-APO was overflowed in the presence or absence of 200 μM NSA.


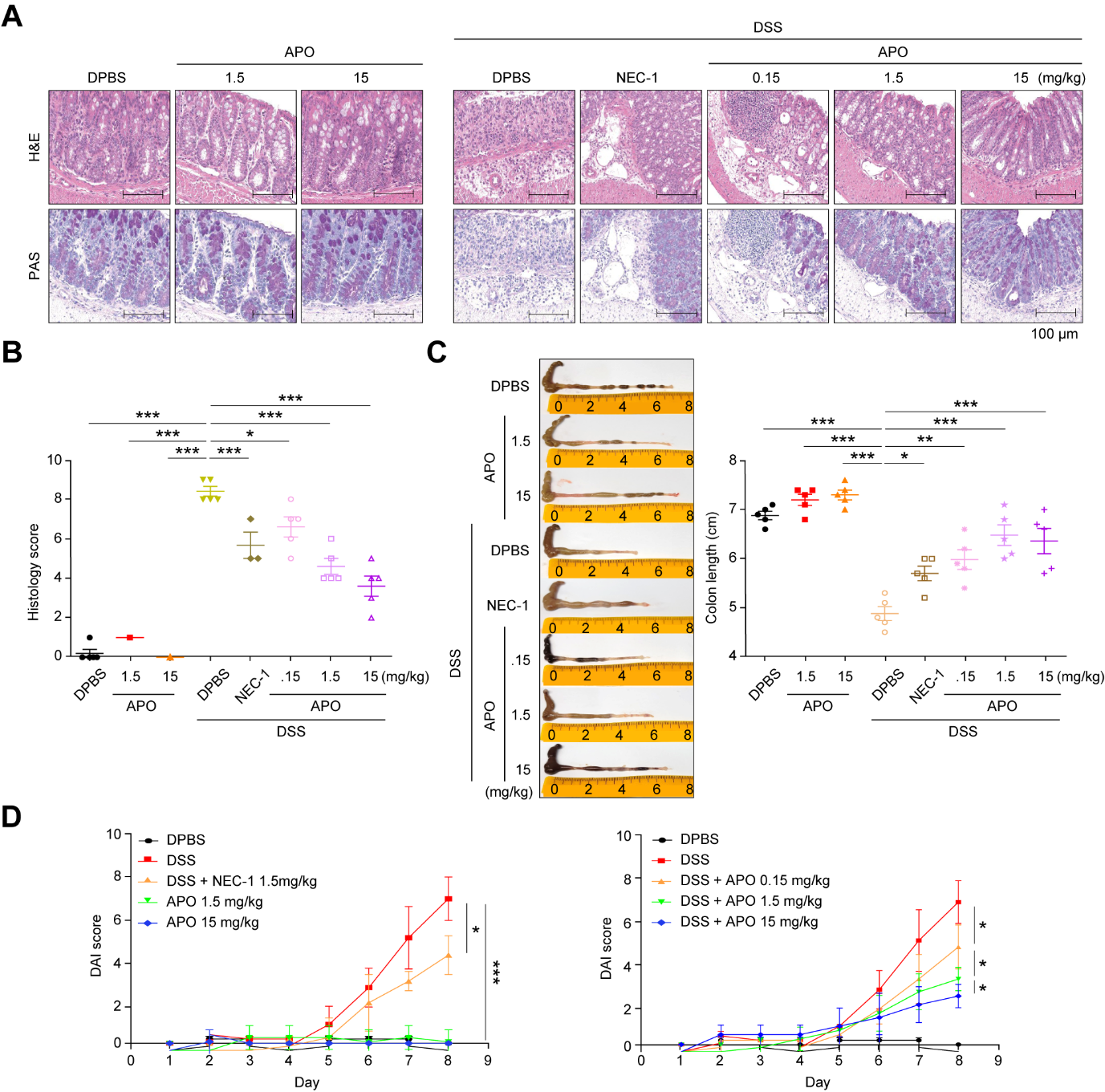


**Fig. S5 APO alleviates the symptoms of inflammatory bowel disease (IBD) in dextran sulfate sodium (DSS)-induced colitis.** (A) As shown in the mouse study design (Fig. 5A), the mouse study was conducted with APO, which was not treated with DTT or oxidation. Mouse colon tissues were collected on day 8, and the effect of APO on colon histopathology was evaluated by H&E and PAS staining. NEC-1 (1.5 mg/kg) was used as a positive control. (B) Histopathologic scores of each group were evaluated. Mean ± SD (n = 5). **p* < 0.05, ****p* < 0.001, one-way ANOVA. (C) Colon length was measured. Representative colon images (left) and colon length (right) are shown. Means ± SD (n = 5). **p* < 0.05, ***p* < 0.01, ****p* < 0.001, one-way ANOVA. (D) Disease activity index (DAI) of colitis was evaluated. Mean ± SD (n = 5). **p* < 0.05, ****p* < 0.001, t-test.


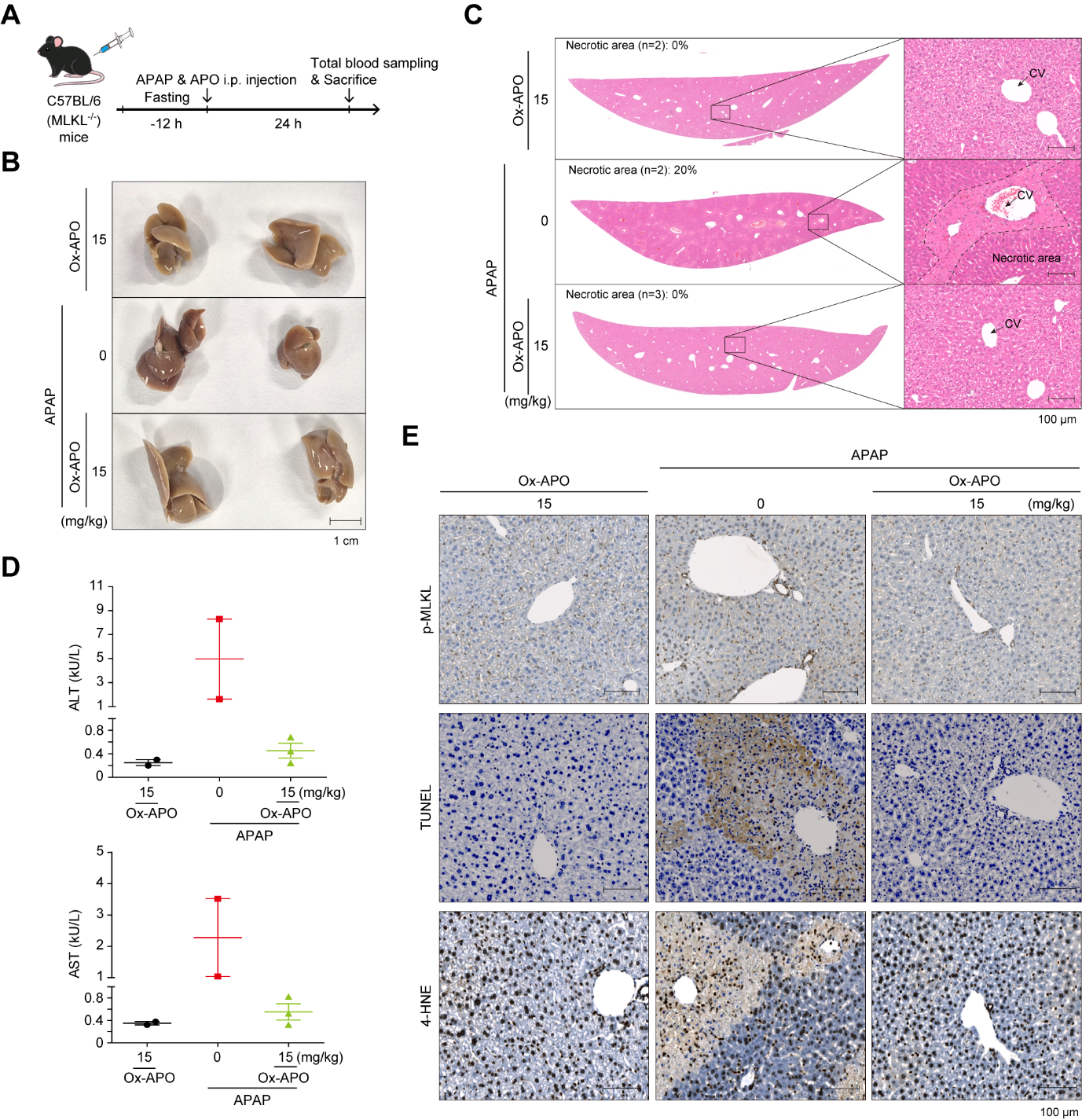


**Fig. S6 APO reduces liver damage in MLKL^–/–^ mice.** (A) MLKL^–/–^ C57BL/6 male mice (6-8 weeks old) received an i.p. injection of 400 mg/kg APAP in the presence or absence of Ox-APO after a 12 h fasting period. Livers were perfused and harvested 24 h after the injection. (B) Gross morphology of liver tissues after APAP injection to induce liver injury. (C) H&E staining of liver tissues after APAP injection. Necrotic areas were quantified as the proportion of unstained regions relative to the total tissue area. The boxed images are magnifications. (D) Serum AST and ALT levels were evaluated to assess liver injury after APAP injection. (E) Representative IHC staining of liver sections for p-MLKL, TUNEL, and 4-HNE. Analyses were performed on paraffin-embedded tissues.

**Supplementary Table**

**Table S1. 72 selected compound candidates**

| **Serial No** | **Compound name** | **^#^% Inhibition** | **Serial No** | **Compound name** | **% Inhibition** |
| --- | --- | --- | --- | --- | --- |
| DPBS | - | 0 | *S38 | Parthenolide | 0 |
| TBZ | - | 100 | S39 | AT7519 | 19.5 |
| - | Necrostatin-1 | 1.2 | S40 | GW3965 | 11.6 |
| *S1 | JP-1302 | 0.4 | S41 | GW 501516 | 9.1 |
| S2 | Neflamapimod | 6.8 | S42 | Rucaparib | 9.1 |
| *S3 | Tyrphostin | 7.5 | S43 | Apatinib | 3.4 |
| *S4 | Oxyphenbutazone | 0 | S44 | Purvalanol A | 3.8 |
| S5 | Fidaxomicin | 5.9 | S45 | CHIR 124 | 6.5 |
| S6 | Z-Leu-Leu-Leu-al | 7.5 | S46 | Tivozanib | 9 |
| S7 | A 784168 | 4.2 | S47 | 5-Iodotubercidin | 4.9 |
| S8 | Bardoxolone methyl | 0 | *S48 | Proscillaridin | 6.8 |
| S9 | Temsirolimus | 19 | S49 | EF-24 | 10.2 |
| *S10 | Pazopanib | 9.4 | S50 | Pranlukast | 10.9 |
| S11 | JTE 013 | 5.8 | S51 | Doramapimod | 3.9 |
| S12 | CVT-313 | 4.7 | S52 | T0901317 | 7.2 |
| S13 | Emetine dihydrochloride | 3.3 | S53 | Exelderm | 8.7 |
| S14 | Cabozantinib | 0.7 | S54 | Quinestrol | 8.2 |
| *S15 | Nitazoxanide | 1.2 | S55 | Proflavine hemisulfate | 3.5 |
| *S16 | Bardoxolone | 0 | S56 | Sirolimus | 0.3 |
| *S17 | Ponatinib | 4.5 | S57 | Pirarubicin | 6.4 |
| *S18 | Ibrutinib | 9.2 | *S58 | Mitoxantrone hydrochloride | 7.6 |
| *S19 | N-(4-Bromophenyl)-3-[[(4-bromophenyl)amino]sulfonyl]benzamide | 5.3 | S59 | Trometamol | 11 |
| S20 | Linifanib | 0 | S60 | Nicardipine hydrochloride | 3.8 |
| S21 | NQ301 | 4.1 | S61 | Daunorubicin | 6 |
| S22 | Sotrastaurin | 4.7 | *S62 | Apomorphine | 7.1 |
| S23 | Prezista | 11.2 | S63 | Cilnidipine | 11 |
| S24 | Ulipristal | 14 | S64 | Lomerizine hydrochloride | 5.3 |
| S25 | 264364 | 1.9 | S65 | Topotecan hydrochloride | 5.2 |
| S26 | Ethacrynic acid | 3 | S66 | Doxorubicin | 8.6 |
| S27 | GW4064 | 9.2 | S67 | Oxiconazole nitrate | 7.1 |
| *S28 | AZD 5438 | 0 | S68 | Fasudil hydrochloride | 6 |
| S29 | STK529529 | 2.8 | S69 | Chloramphenicol | 0 |
| S30 | Vemurafenib | 6.4 | S70 | Diphenhydramine | 19.1 |
| *S31 | Dabrafenib | 4.5 | S71 | PPT | 1.5 |
| S32 | SB 268262 | 0 | *S72 | Niclosamide | 0.9 |
| S33 | STK528708 | 0.1 |  |  |  |
| *S34 | 267254 | 7.8 |  |  |  |
| *S35 | Costunolide | 9 |  |  |  |
| S36 | AR-A014418 | 10.8 |  |  |  |
| S37 | Motesanib | 9.4 |  |  |  |

#: % inhibition that reduced luciferase activity to below 20% of the TBZ-treated supernatant from 2150 compounds.

*: Eighteen selected compound candidates were selected for next screening assay after the p-MLKL Western blot.
