# Supplementary material for: Apomorphine is a novel necroptosis inhibitor targeting mixed lineage kinase domain-like protein oligomerization
Source: Cell Death Discov. 2025 Oct 13;11:457. doi: 10.1038/s41420-025-02763-8 (PMC12518639; doi:10.1038/s41420-025-02763-8)
Supplement: Supplementary file 2 — Unedited blot and gel images [file 41420_2025_2763_MOESM2_ESM.docx]

**Figure 1C**


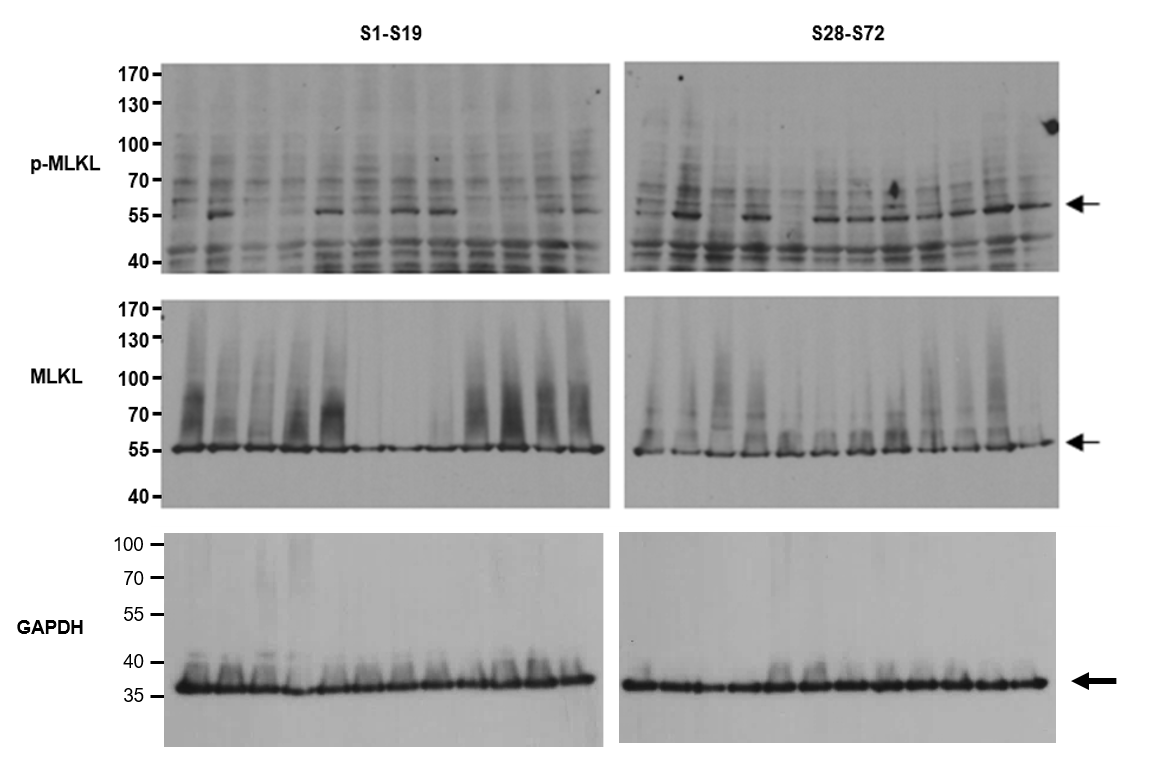


**Figure 1D**


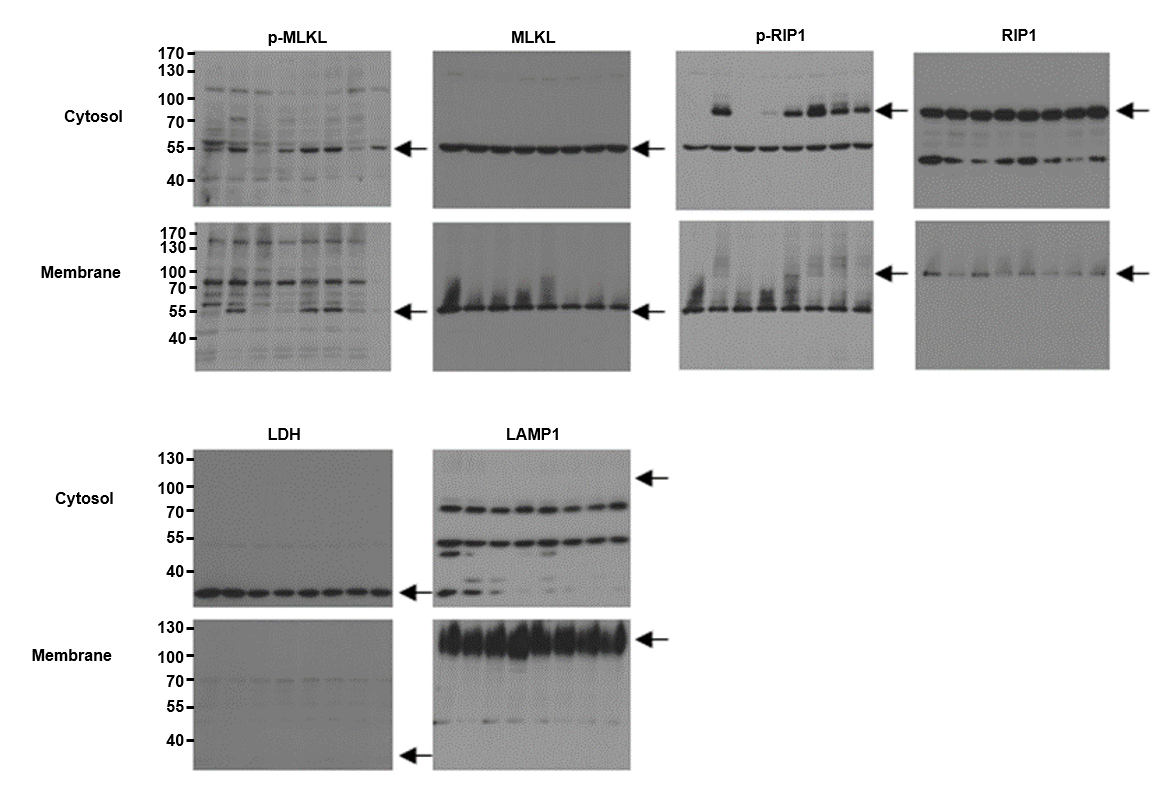


**Figure 1E**


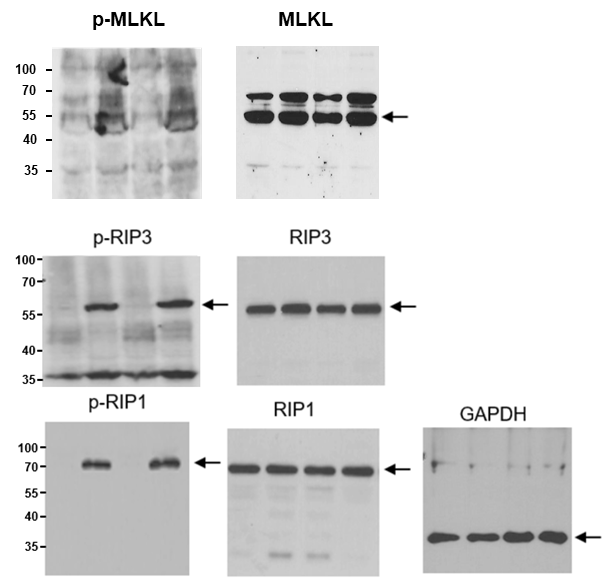


**Figure 1G**


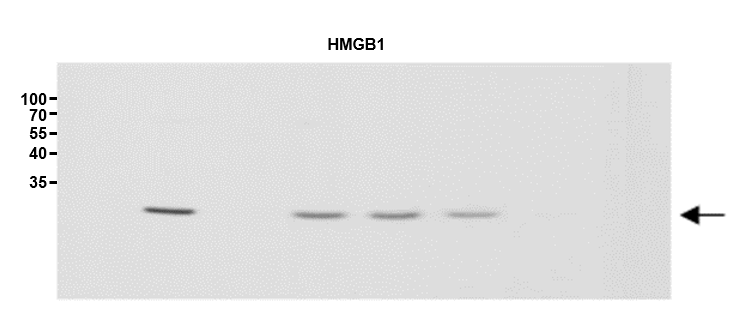


**Figure 2C**

Non-reducing condition


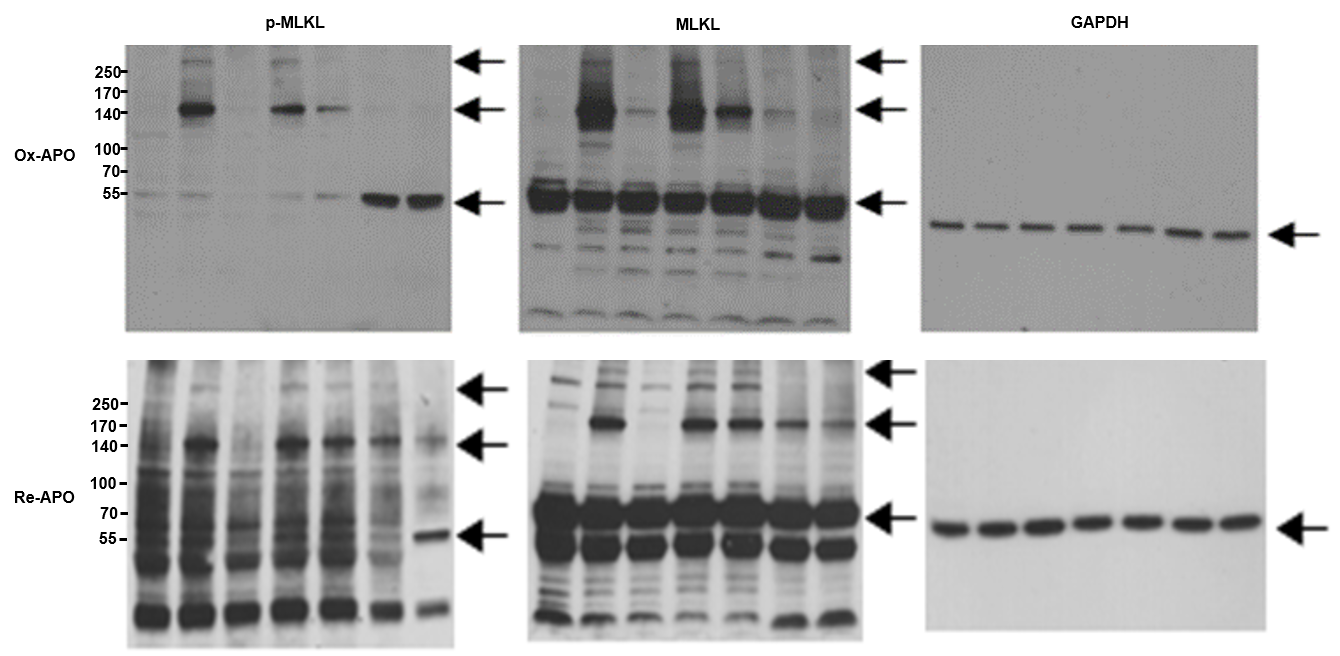


Reducing condition

Re-APO Ox-APO


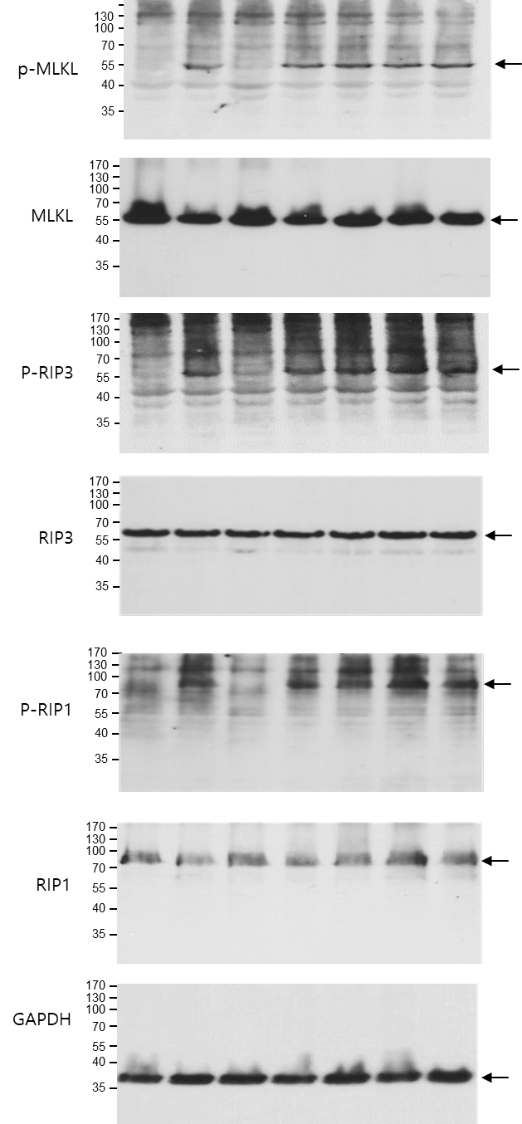

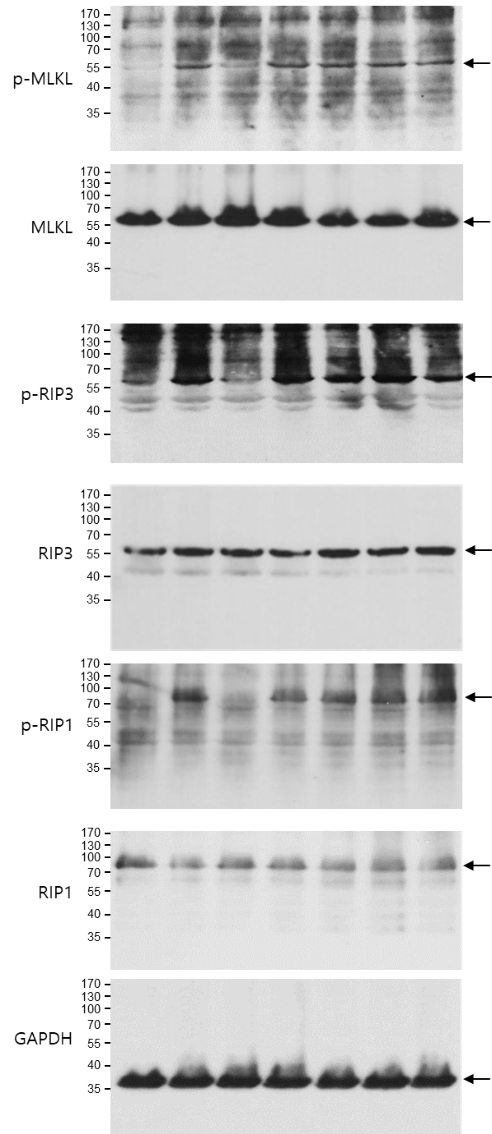


**Figure 2E**

Non-reducing condition


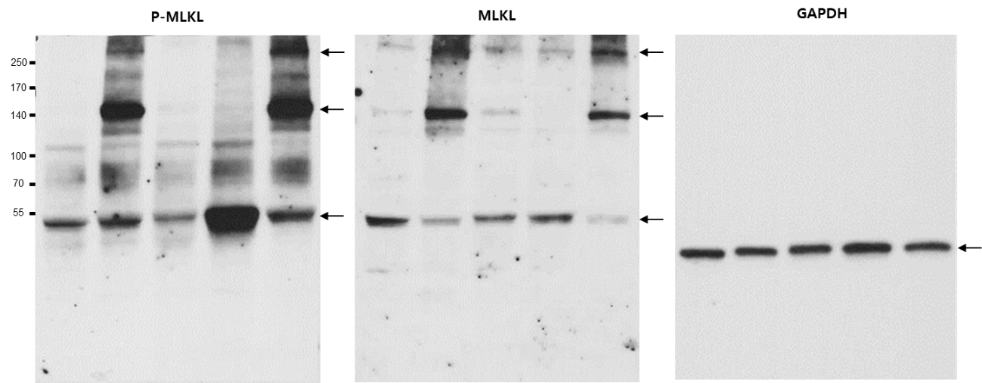


Reducing-condition


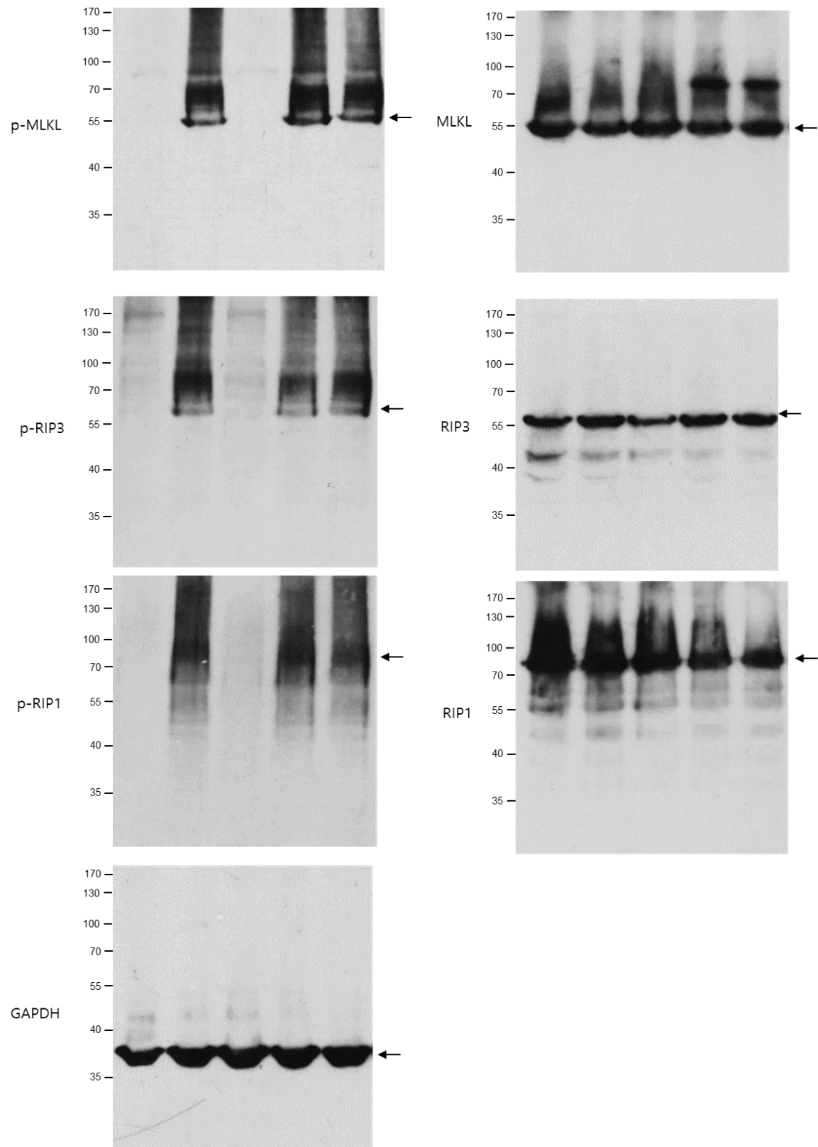


**Figure S1**

**
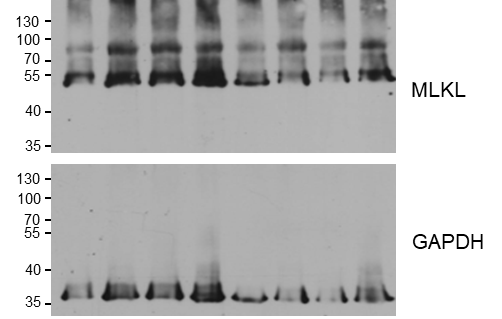
**
